# Galantamine–Escitalopram Combination Therapy in Alzheimer’s Comorbid Depression Model in Mice: Role of BDNF/KYN Pathways, Neuroinflammation, and Oxidative Stress

**DOI:** 10.1021/acsomega.5c08276

**Published:** 2026-01-28

**Authors:** Shivanshu Bajaj, Radhakrishnan Mahesh

**Affiliations:** Department of Pharmacy, 29794 Birla Institute of Technology & Science (BITS), Pilani Campus, Pilani, Rajasthan 333031, India

## Abstract

Alzheimer’s disease (AD) is the most prevalent form of dementia, accounting for more than two-thirds of cases in older adults. AD is associated with neuropsychiatric symptoms such as depression, anxiety, and sleep disturbances. The coexistence of AD with depression, in particular, poses serious challenges and often results in suboptimal outcomes with conventional therapies. The present study therefore aimed to investigate the therapeutic potential of escitalopram (ESC; SSRI) in combination with galantamine (GAL; AChE inhibitor) on key pathological pathways, including the neurotrophic system, hypothalamic–pituitary–adrenal (HPA) axis, kynurenine pathway, inflammation, and oxidative stress, in an animal model of AD comorbid with depression. Swiss albino mice were subjected to chronic mild stress (CMS) for 21 days and received intrahippocampal administration of amyloid-β peptide to mimic AD-depression comorbidity. Subsequently, ESC (10 mg/kg) combined with GAL (5 mg/kg) was administered orally for 20 days alongside the CMS protocol, followed by behavioral, biochemical, and histopathological assessments. The combined GAL + ESC treatment significantly alleviated depressive symptoms and improved working and spatial memory in CMS and amyloid-β-exposed mice. Furthermore, the therapy normalized hippocampal levels of BDNF, proinflammatory cytokines (IL-6, TNF-α), kynurenine metabolites (3-HK, QUIN), and oxidative stress markers toward those observed in the sham group. Histopathological analysis further confirmed the preservation of hippocampal integrity with combined therapy. Overall, the findings highlight the potential of ESC as an adjunct to GAL in ameliorating depressive symptoms and cognitive deficits, underscoring its promise for further clinical evaluation in the management of AD comorbid with depression.

## Graphical abstract

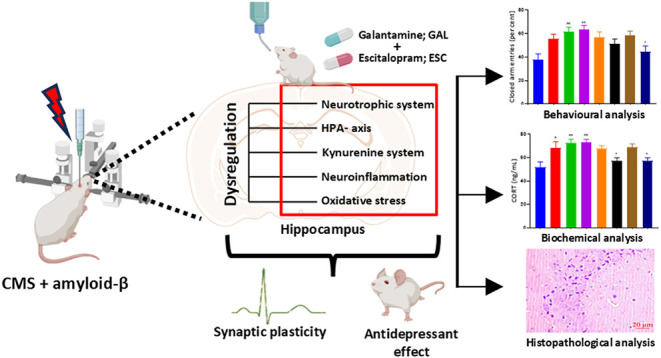

## 1 Introduction

Alzheimer’s disease (AD) is the most prevalent form of dementia and the seventh leading cause of mortality among older adults, accounting for 60–70% of global dementia cases. According to the World Health Organization (WHO), an estimated 57 million people were living with dementia worldwide in 2021, with nearly 10 million new cases reported annually.^1^ AD is generally associated with cognitive impairment and related affective symptoms, including depression, anxiety, sleep disturbances, psychosocial disability, irritability, paranoia, and others. A meta-analysis projected the pooled prevalence of neuropsychiatric symptoms across all stages of AD, highlighting serious complications. The most common symptoms included apathy (49%), followed by depression (42%), aggression (40%), anxiety (39%), and sleep disturbances (39%).^2^ Depression functions as a significant accelerating factor in the clinical trajectory of AD, influencing its progression from mild cognitive impairment to advanced dementia. Furthermore, the distinct characteristics of depression in the early versus late stages of AD remain partially understood, primarily due to the diagnostic challenges posed by the complexity of identifying AD-comorbid depression.^3^

AD and depression share an exceedingly complex pathophysiology, involving multiple interrelated mechanisms such as disrupted neurotrophic signaling, hypothalamic–pituitary–adrenal (HPA) axis dysregulation, phosphorylated tau and amyloid-β accumulation, and alterations in key neurotransmitters, including acetylcholine (ACh), serotonin, gamma-aminobutyric acid (GABA), norepinephrine, and dopamine.^4^ Neurotrophins, particularly brain-derived neurotrophic factor (BDNF), are synthesized throughout adult life and play a crucial role in maintaining neuronal homeostasis and regulating synaptic plasticity. In disease pathogenesis, BDNF levels are markedly reduced in both AD and depression, especially in the hippocampus and prefrontal cortex regions, which are known for their critical functions in cognition and mood regulation. Decreased BDNF levels are associated with impaired synaptic plasticity, reduced neurogenesis, increased inflammation, and oxidative stress.^3^ Inflammation is another shared hallmark of these conditions, often correlated with disease severity. Chronic stress and amyloid-β exposure can lead to increased levels of pro-inflammatory cytokines, such as interleukin-6 (IL-6) and tumor necrosis factor-alpha (TNF-α), primarily through aberrant glucocorticoid signaling and microglial activation. Sustained elevation of pro-inflammatory cytokines contributes to neurodegeneration, cognitive impairment, increased reactive oxygen species (ROS), apoptosis, and neurotoxicity.^5^ The HPA axis also plays a key role in linking stress and brain function, and its dysregulation is a hallmark of several psychiatric disorders. Numerous studies have demonstrated that early life stress contributes to long-term neurobiological changes, including those associated with AD and depression, often characterized by elevated corticosterone (CORT) (or cortisol in humans) and HPA axis dysfunction.^6^ Dysregulation of the kynurenine pathway represents a common pathological mechanism in both AD and depression. This pathway governs the central and peripheral catabolism of the essential amino acid L-tryptophan. An imbalance between these neuroprotective metabolites (kynurenine and kynurenic acid) and neurotoxic metabolites (quinolinic acid (QUIN) and 3-hydroxykynurenine (3-HK)) enhances neurotoxicity, oxidative stress, glutamate excitotoxicity, hippocampal atrophy, cognitive dysfunction, emotional dysregulation, and neurodegeneration, which contribute to the pathophysiology of AD and depression.^7^ Understanding the role of the kynurenine pathway in AD comorbid depression is an emerging area of research that highlights the intersection of multiple dysregulated mechanisms in this complex pathology. A comprehensive understanding of these shared molecular pathways is essential for developing novel, targeted therapeutic strategies to address the multifaceted nature of this comorbidity.

To replicate the dual pathology of AD and depression in animals, we employed a combination of chronic mild stress (CMS) and intrahippocampal administration of amyloid-β (Aβ_1–42_) in Swiss albino mice. CMS models early life stressors, a major contributor to depression pathology, while Aβ_1–42_ oligomers induce neuropathological changes characteristic of AD. The concurrent application of these insults simulates a comorbid condition comparable to the human disease state, thereby providing a platform to investigate shared neurobiological mechanisms and evaluate potential therapeutic strategies.

Currently, there are no approved treatments or established clinical guidelines specifically for managing AD-comorbid depression. FDA-approved therapies primarily target individual diseases. Likewise, AD includes acetylcholinesterase enzyme (AChE) inhibitors (galantamine (GAL), donepezil, and rivastigmine); *N*-methyl-d-aspartate (NMDA) receptor antagonists (memantine); and monoclonal antibodies (aducanumab and lecanemab). Pharmacological treatment options for depression include selective serotonin reuptake inhibitors (SSRIs; escitalopram (ESC), citalopram, fluoxetine, and sertraline) and serotonin-norepinephrine reuptake inhibitors (SNRIs; desvenlafaxine, duloxetine, and venlafaxine). Treating patients with AD-comorbid depression remains clinically challenging due to the limited efficacy of available pharmacotherapies. Monotherapy with either antidepressants (SSRIs or SNRIs) or AChE inhibitors is often suboptimal, with limitations such as complex pathophysiology, inadequate therapeutic response, increased risk of adverse effects, and the development of drug tolerance.^3^ To overcome these challenges, combination therapy has emerged as a promising approach, offering the potential for improved clinical outcomes.^8^ There is increasing interest in evaluating the concurrent use of SSRI agents with AChE inhibitors for the treatment of AD-comorbid depression.^9^ This integrative pharmacological strategy aims to achieve greater therapeutic efficacy than conventional monotherapy.^10,11^

In this study, emphasis was placed on the combination of two drugs: GAL and ESC. GAL, an FDA-approved AChE inhibitor indicated for the treatment of mild to moderate AD, has shown potential therapeutic benefits in psychiatric disorders such as schizophrenia, major depressive disorder (MDD), bipolar disorder, and alcohol dependence.^12^ Although considered a relatively weak AChE inhibitor, GAL exerts a unique allosteric potentiating effect on α7 nicotinic acetylcholine receptors (α7 nAChRs). This mechanism not only enhances cholinergic neurotransmission but also indirectly modulates other neurotransmitter systems, including monoamines, glutamate, and GABA. Moreover, muscarinic receptor activation has been implicated in the antipsychotic-like effects of GAL.^13^ When administered alongside established antidepressants, GAL has demonstrated antidepressant-like properties, likely through modulation of key neurobiological systems such as the HPA-axis, dopaminergic signaling, inflammatory cascades, and oxidative stress.^14^ Owing to these psychotropic properties, GAL is hypothesized to complement ESC, an FDA-approved SSRI used in the treatment of unipolar depression and generalized anxiety disorder. ESC enhances serotonin availability in the synaptic cleft and interacts with multiple serotonin receptor subtypes, thereby modulating mood, cognition, and emotional regulation.^15,16^ The combination of GAL and ESC was therefore anticipated to exert complementary and synergistic effects, targeting both cholinergic and serotonergic systems and ultimately offering greater therapeutic efficacy in AD comorbid with depression than either agent alone.^17^ Moreover, it was hypothesized that the GAL-ESC combination would more effectively modulate impaired neurobiological signaling compared to monotherapy by ameliorating kynurenine pathway metabolites, enhancing neurotrophic markers, and attenuating inflammation and oxidative stress, thereby substantially improving the comorbid disease state.^9,11,18^

To the best of our knowledge, there are currently no published reports that utilize a combination of CMS and Aβ_1–42_ to establish an *in vivo* mouse model of AD-comorbid depression. This study aimed to evaluate the effects of a combination therapy of GAL (AChE inhibitor) and ESC (SSRI) using the comorbid model, with a focus on delineating alterations in neurotrophic and kynurenine signaling, the HPA axis, neuroinflammation, oxidative stress, and cognitive function. The findings from this work are expected to contribute to a deeper understanding of the pathophysiology of AD-comorbid depression and the associated molecular and behavioral changes.

## 2 Results

### 2.1 GAL and ESC Combination Attenuated Depressive Behavior in Alzheimer’s-Comorbid Depression Model in Mice

Elevated plus maze (EPM)-based behavioral test was performed to evaluate the effect of the GAL + ESC combination in the AD-comorbid depression model. The Aβ_1–42_ + CMS group exhibited a significant increase in % closed arm entries (63.53 ± 3.36% vs 38.13 ± 4.68%, *p* < 0.01) and reduced % open arm entries (18.47 ± 3.30% vs 45.87 ± 4.68%, *p* < 0.001) compared to the sham group, indicating depression-like behavior in mice. Treatment with COMB­(II) resulted in a marked reduction in the % closed arm entries (44.87 ± 4.60%; *p* < 0.05) compared to the Aβ_1–42_ + CMS group, whereas COMB­(I), GAL, and ESC exhibited nonsignificant changes in % closed arm (58.60 ± 3.64%; *p* = 0.98, 57.08 ± 4.5%; *p* = 0.95 and 51.35 ± 4.12%; *p* = 0.42) compared to the Aβ_1–42_ + CMS group, respectively (Figure 1B).

**Figure 1:**
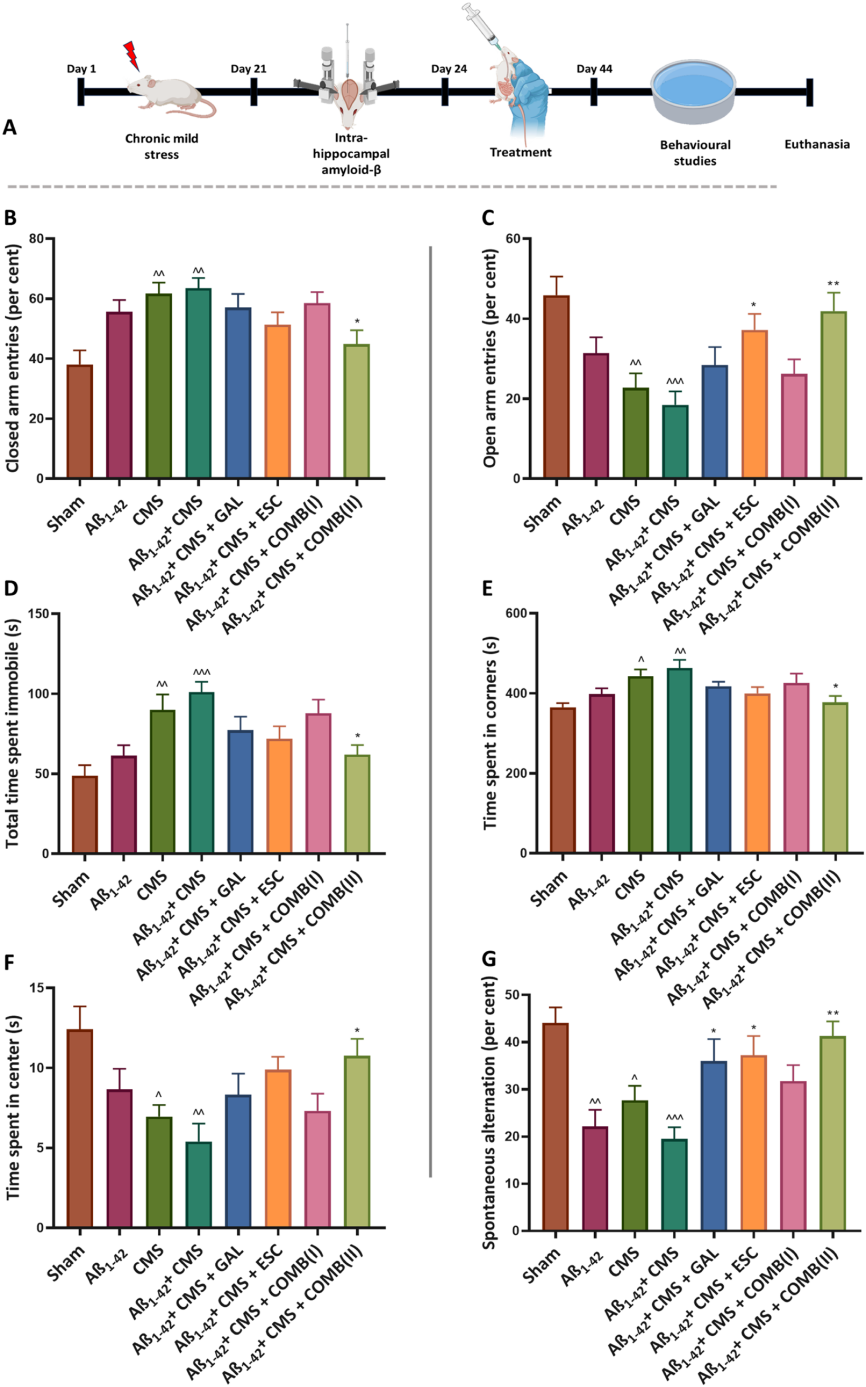
Effect of GAL, ESC, and combination in behavioral tests against the Aβ_1–42_ + CMS-induced model of AD-comorbid depression. A) The study design. B) and C) % closed and % open arm entries in EPM. D) Total time spent immobile in FST. E) and F) Time spent by the mice in the corner and center of OFT. G) % spontaneous alternation behavior. The results were statistically analyzed by one-way ANOVA followed by the posthoc Tukey–Kramer multiple comparison test. The values were presented as mean ± SEM (*n* = 8). Statistical significance is marked as ^^^*p* < 0.001, ^^*p* < 0.01, ^*p* < 0.05, compared to the sham group; **p < 0.01 and *p < 0.05, compared to the Aβ_1–42_ + CMS group. Doses: GAL (5 mg/kg), ESC (10 mg/kg), COMB­(I) (GAL; 3 mg/kg + ESC; 5 mg/kg), COMB­(II) (GAL; 5 mg/kg + ESC; 10 mg/kg).

Moreover, treatment with COMB­(II) and ESC showed a significant increase in % open arm entries (41.93 ± 4.60; *p* < 0.01 and 37.23 ± 3.98; *p* < 0.05) compared to the Aβ_1–42_ + CMS group (18.47 ± 3.30).

While COMB­(I) and GAL exhibited nonsignificant changes in % open arm entries (26.21 ± 3.64; *p* = 0.88 and 28.42 ± 4.51; *p* = 0.67), respectively, compared to the Aβ_1–42_ + CMS group (Figure 1C), the one-way ANOVA showed large effect sizes for % closed arm entries (η^2^ = 0.35) and % open arm entries (η^2^ = 0.40), suggesting that treatment conditions exerted a strong influence on EPM performance.

The forced swim test (FST) was used to measure behavioral despair, a prominent method for screening antidepressants. In the study, the Aβ_1–42_ + CMS group showed a significant increase in total immobility time (101.2 ± 6.36 s; *p* < 0.001) compared to the sham group (48.79 ± 6.62 s). Treatment with COMB­(II) resulted in a decrease in time spent immobile (62.08 ± 5.85 s; *p* < 0.05) compared to the Aβ_1–42_ + CMS group. In comparison, COMB­(I), GAL, and ESC exhibited nonsignificant changes in time spent immobile (87.85 ± 8.55 s; *p* = 0.91, 77.26 ± 8.48 s; *p* = 0.34 and 72.0 ± 7.69 s; *p* = 0.13), compared to the Aβ_1–42_ + CMS group (Figure 1D). In the FST, the one-way ANOVA revealed a significant effect of treatment on total immobility time, with a large effect size (η^2^ = 0.39), indicating that treatment accounted for a substantial proportion of the variance in despair-like behavior.

The open field test (OFT) is another behavioral test used to measure anxiety-like behavior related to depression in mice. The Aβ_1–42_ + CMS group showed a significant increase in time spent (s) in the corner (463.5 ± 20.53 s vs 365.1 ± 10.31 s; *p* < 0.01) and reduced time spent in the center of the arena (5.4 ± 0.71 s vs 12.41 ± 1.43 s; *p* < 0.01) compared to the sham group. The treatment with COMB­(II) resulted in a marked reduction in time spent in corners (377.6 ± 15.90 s; *p* < 0.05) compared to the Aβ_1–42_ + CMS group, whereas treatment with COMB­(I), GAL, and ESC exhibited nonsignificant changes in time spent in the corner (426.1 ± 23.55 s; *p* = 0.75, 417.8 ± 11.04 s; *p* = 0.52 and 399.9 ± 15.99 s; *p* = 0.14) compared to the Aβ_1–42_ + CMS group (Figure 1E).

Subsequently, treatment with COMB­(II) resulted in an elevated time spent in the center compartment (10.76 ± 1.05 s; *p* < 0.05) compared to the Aβ_1–42_ + CMS group. In contrast, COMB­(I), GAL, and ESC exhibited nonsignificant changes in time spent in the center (7.32 ± 1.07 s; *p* = 0.92, 8.32 ± 1.32 s; *p* = 0.60 and 9.8 ± 0.81 s; *p* = 0.11), compared to the Aβ_1–42_ + CMS group (Figure 1F). The one-way ANOVA for the OFT demonstrated significant treatment effects on both time spent in the corner and center zones, each with large effect sizes (η^2^ = 0.33), indicating that treatment groups explained a considerable portion of the variance in exploratory and anxiety-like behaviors.

### 2.2 GAL and ESC Combination Improved Cognitive Dysfunction in the Alzheimer’s-Comorbid Depression Model

#### 2.2.1 Spontaneous Alternation Behavior (SAB) Test

The test was used to evaluate the spatial working memory in rodents. The Aβ_1–42_ + CMS group showed a significant decrease in % spontaneous alternation (SA) behavior (19.49 ± 2.48%; *p* < 0.001) compared to the sham group (44.06 ± 3.34%). The treatment with GAL and ESC alone normalized % SA (35.98 ± 4.67%; *p* < 0.05 and 37.26 ± 4.06%; *p* < 0.05) compared to the Aβ_1–42_ + CMS group. However, treatment with COMB­(II) resulted in a statistically significant increase in % SA (41.28 ± 3.10%; *p* < 0.01) compared to the Aβ_1–42_ + CMS group, whereas COMB­(I) exhibited nonsignificant changes in % SA (31.79 ± 3.35%; *p* = 0.22) compared to the Aβ_1–42_ + CMS group (Figure 1G). The one-way ANOVA revealed a large effect size for % SA (η^2^ = 0.44), indicating that treatment had a strong influence on cognitive performance.

#### 2.2.2 Morris Water Maze (MWM) Test

The test was employed to evaluate spatial learning and memory in rodents. The test consists of two main phases: the acquisition phase and the probe trial.

#### 2.2.3 Spatial Acquisition Test

Figure 2A shows the mean escape latency to find the hidden platform during 5 days of training. The Aβ_1–42_ + CMS group showed a significant increase in the escape latency (52.46 ± 1.33 s; *p* < 0.001) compared to the sham group (22.98 ± 2.42 s). The treatment with COMB­(II) showed a significant reduction in escape latency (25.82 ± 3.25 s; *p* < 0.001) compared to the Aβ_1–42_ + CMS group. COMB­(I) exhibited a nonsignificant decrease in escape latency (39.56 ± 2.65 s; *p* = 0.13) compared to the Aβ_1–42_ + CMS group. Moreover, GAL and ESC normalized the escape latency (39.40 ± 3.13 s; *p* < 0.05 and 38.79 ± 3.74 s; *p* < 0.05), compared to the Aβ_1–42_ + CMS group (Figure 2A). Repeated-measures ANOVA for escape latency in the MWM demonstrated significant main effects of treatment (column factor: ηp^2^ = 0.68) and time (ηp^2^ = 0.29), as well as a moderate treatment × time interaction (ηp^2^ = 0.18), indicating robust treatment-dependent improvements in spatial learning across trials. During 5 days of training trials, a significant increase in the path length (meters; m) was observed in the Aβ_1–42_ + CMS group (26.80 ± 0.65 m; *p* < 0.01) compared to the sham group (17.68 ± 2.29 m). Upon treatment with COMB­(II), GAL, and ESC, path length was significantly reduced on day 5 (19.06 ± 2.46 m; *p* < 0.05, 20.61 ± 2.32 m; *p* < 0.05 and 20.12 ± 2.34; *p* < 0.05), compared to the Aβ_1–42_ + CMS group (Figure 2B). Repeated-measures ANOVA for path length showed significant main effects of treatment (ηp^2^ = 0.37) and time (ηp^2^ = 0.47), as well as a moderate treatment × time interaction (ηp^2^ = 0.14), indicating substantial treatment-dependent improvements in spatial navigation and learning dynamics. No significant difference in the mean speed was observed among the drug-treated groups and the Aβ_1–42_ + CMS group, indicating no effect on the motor activity of mice (Figure 2C).

**Figure 2:**
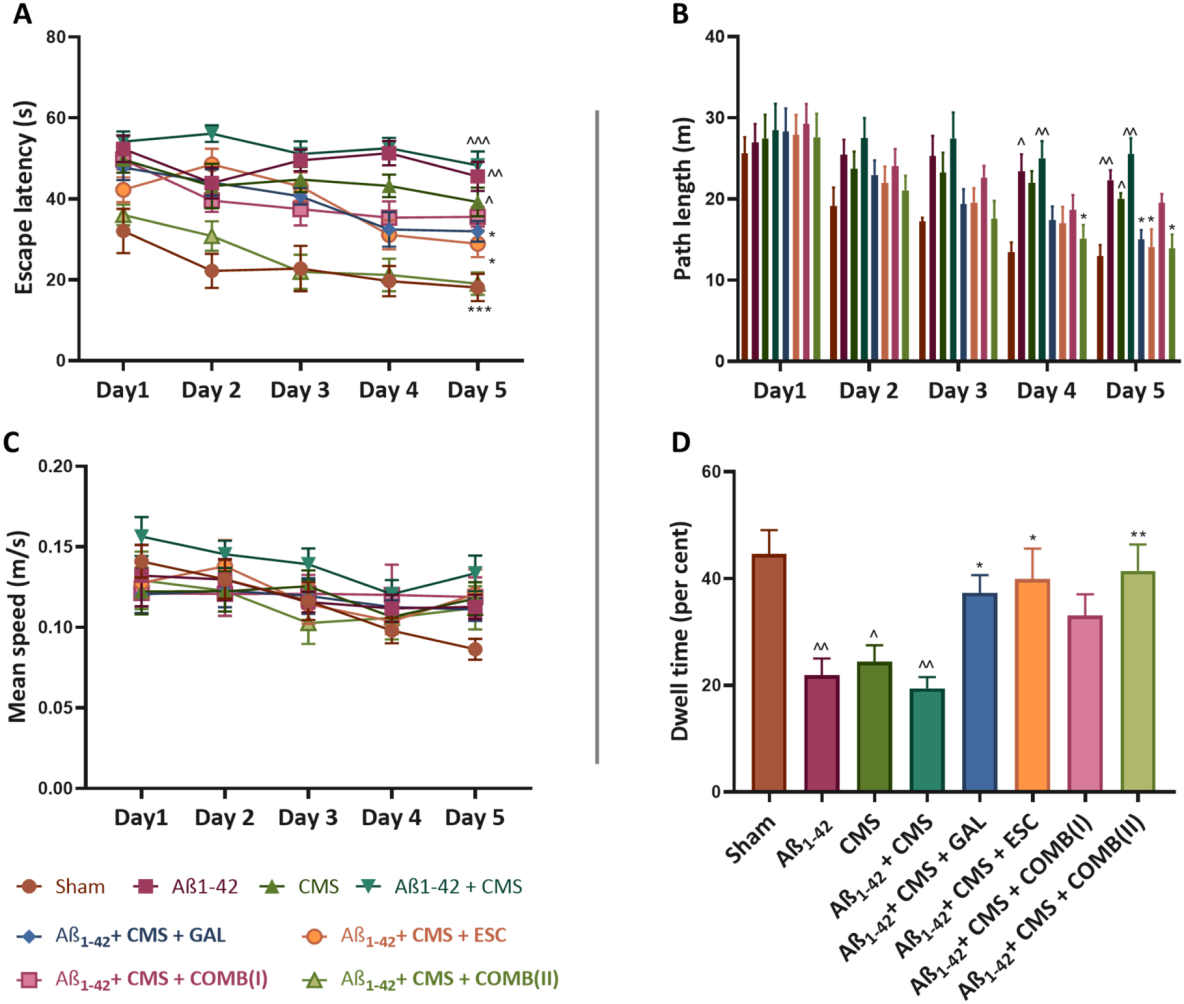
GAL, ESC, and combination ameliorated cognitive dysfunction in the Aβ_1–42_ + CMS-induced model of AD-comorbid depression. A) Escape latency, B) path length, C) mean speed, and D) % dwell time in MWM. The results were statistically analyzed by repeated measure ANOVA for escape latency, path length, and mean speed followed by the posthoc Tukey–Kramer multiple comparison test and one-way ANOVA for % dwell time. The values were presented as mean ± SEM (*n* = 8). Statistical significance is marked as ^^^*p* < 0.001, ^^*p* < 0.01, ^*p* < 0.05, compared to the sham group; ***p < 0.001, **p < 0.01, and *p < 0.05, compared to the Aβ_1–42_ + CMS group. Doses: GAL (5 mg/kg), ESC (10 mg/kg), COMB­(I) (GAL; 3 mg/kg + ESC; 5 mg/kg), COMB­(II) (GAL; 5 mg/kg + ESC; 10 mg/kg).

#### 2.2.4 Reference Memory Test

After the spatial acquisition trial was completed, 24 h later, the probe trial was performed. The Aβ_1–42_ + CMS group failed to memorize the location of the platform, as shown by a significantly lower % dwell time in the target quadrant (19.44 ± 2.10%; *p* < 0.01) compared to the sham group (44.61 ± 4.46%) (Figure 2D). The treatment with GAL and ESC alone significantly improved % dwell time (37.31 ± 3.3%; *p* < 0.05 and 39.93 ± 5.69%; *p* < 0.05), compared to the Aβ_1–42_ + CMS group. However, the treatment with COMB­(II) showed a stronger statistical association with increased % dwell time in the target quadrant (41.44 ± 4.95%; *p* < 0.01) compared to the Aβ_1–42_ + CMS group. In contrast, COMB­(I) did not significantly improve % dwell time (33.06 ± 4%; *p* = 0.25) compared to the Aβ_1–42_ + CMS group (Figure 2D). In the probe trial, one-way ANOVA revealed a large effect size for % dwell time in the target quadrant (η^2^ = 0.42), indicating that treatment accounted for a substantial proportion of variance in spatial memory retention. Moreover, in the reference memory test, monotherapy and combined therapy effectively improved the cognitive dysfunction of mice subjected to Aβ_1–42_ + CMS.

### 2.3 GAL and ESC Combination Ameliorated BDNF Expression in Hippocampus of the Alzheimer’s-Comorbid Depression Model

BDNF is a key protein in the brain that plays an essential role in neural development, connectivity, learning, and memory. BDNF levels were estimated using an ELISA-based assay. The Aβ_1–42_ + CMS group showed a significant reduction in BDNF levels in the hippocampus (686.3 ± 80.8 pg/mL; *p* < 0.01) compared to the sham group (1112 ± 81.34 pg/mL). Treatment with GAL and ESC alone showed improvements in BDNF levels (1019 ± 75.33 pg/mL; *p* < 0.05 and 1017 ± 38.71pg/mL; *p* < 0.05), compared to the Aβ_1–42_ + CMS group. Moreover, treatment with COMB­(II) markedly improved BDNF levels (1094 ± 70.64 pg/mL; *p* < 0.01) compared to the Aβ_1–42_ + CMS group, while COMB­(I) exhibited nonsignificant changes in BDNF levels (802.7 ± 73.8 pg/mL; *p* = 0.92) compared to the Aβ_1–42_ + CMS group (Figure 3A). One-way ANOVA for hippocampal BDNF levels revealed a large effect size (η^2^ = 0.53), indicating that treatment accounted for a substantial proportion of variance in neurotrophic expression. Both monotherapy and combined therapy increased hippocampal BDNF levels in mice subjected to Aβ_1–42_ + CMS; however, relative to monotherapy, the combined therapy yielded a markedly greater elevation in BDNF concentration.

**Figure 3:**
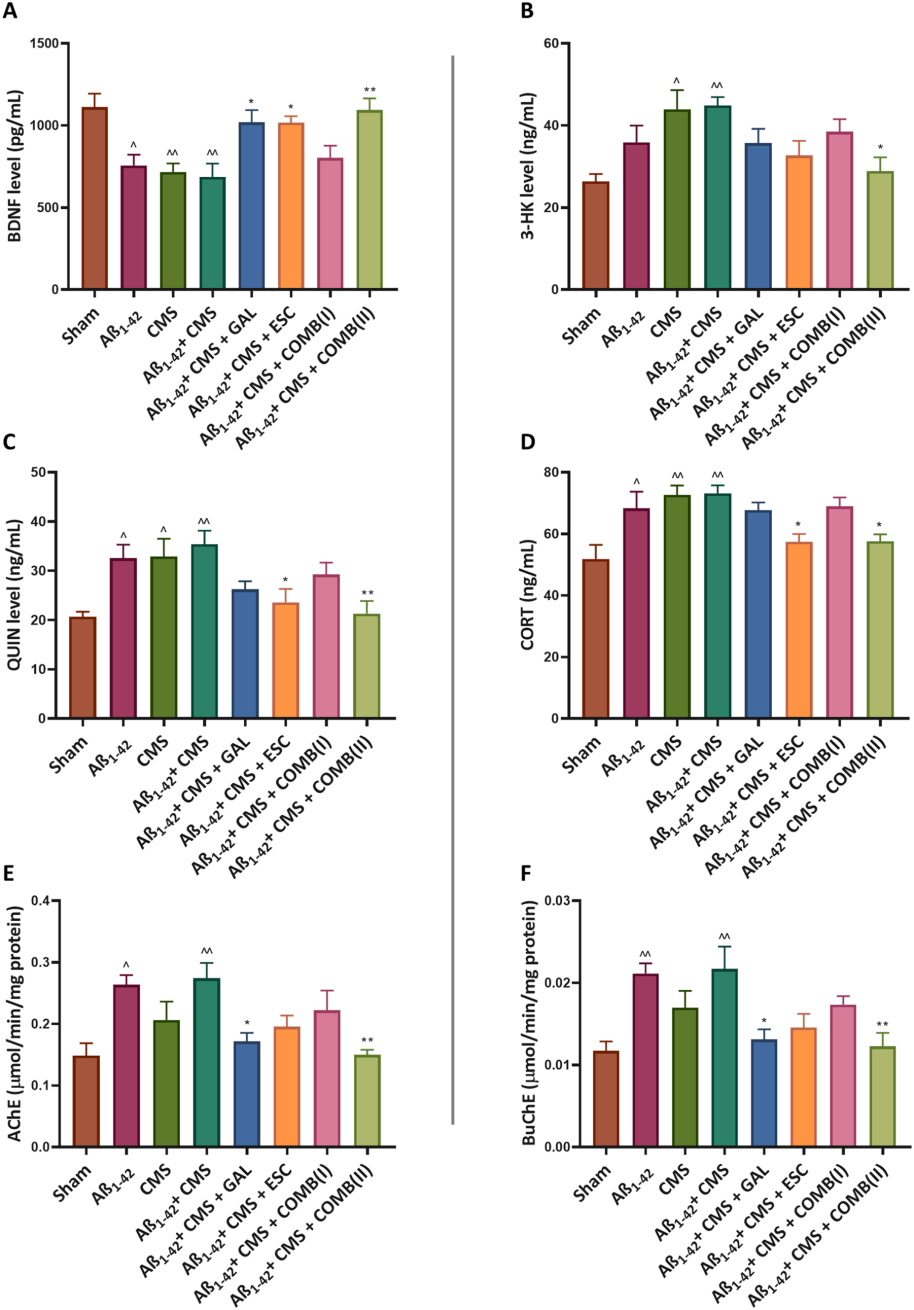
GAL, ESC, and combination normalize the levels of biochemical anomalies induced by the Aβ_1–42_ + CMS-induced model of AD-comorbid depression. A) BDNF levels (pg/mL), B) 3-HK levels (ng/mL), C) QUIN levels (ng/mL), D) CORT levels (ng/mL), E) AChE levels (μmol/min/mg protein), F) BuChE levels (μmol/min/mg protein). The results were statistically analyzed by one-way ANOVA followed by the posthoc Tukey–Kramer multiple comparison test. The values were presented as mean ± SEM (*n* = 6). Statistical significance is marked as ^^*p* < 0.01, ^*p* < 0.05, compared to the sham group; **p < 0.01, and *p < 0.05, compared to the Aβ_1–42_ + CMS group. Doses: GAL (5 mg/kg), ESC (10 mg/kg), COMB­(I) (GAL; 3 mg/kg + ESC; 5 mg/kg), COMB­(II) (GAL; 5 mg/kg + ESC; 10 mg/kg).

### 2.4 GAL and ESC Combination Reduce the Neurotoxicity in Kynurenine Pathway in the Alzheimer’s-Comorbid Depression Model

In the kynurenine pathway, 3-HK is the neurotoxic metabolite that acts as a free radical, causing cellular damage and neuronal death. The Aβ_1–42_ + CMS group showed a significant increase in 3-HK levels in the hippocampus (44.93 ± 2 ng/mL; *p* < 0.01) compared to the sham group (26.36 ± 1.81 ng/mL). The treatment with COMB­(II) significantly lowered the levels of 3-HK (28.89 ± 3.35 ng/mL; *p* < 0.05) compared to the Aβ_1–42_ + CMS group. In contrast, COMB­(I), GAL, and ESC exhibited nonsignificant changes in the 3-HK levels (38.44 ± 3.1 ng/mL; *p* = 0.86, 35.73 ± 3.42 ng/mL; *p* = 0.54 and 32.71 ± 3.53 ng/mL; *p* = 0.20), compared to the Aβ_1–42_ + CMS group (Figure 3B). One-way ANOVA for hippocampal 3-HK levels revealed a large effect size (η^2^ = 0.40), indicating that treatment accounted for a substantial proportion of variance in kynurenine pathway metabolism.

Subsequently, QUIN is another neurotoxic metabolite of tryptophan that dysregulates glutamate signaling, causing excessive neuronal excitation and contributing to the pathology of depression and associated comorbidities. The Aβ_1–42_ + CMS group showed a significant increase in QUIN levels in the hippocampus (35.44 ± 2.72 ng/mL; *p* < 0.01) compared to the sham group (20.67 ± 1.02 ng/mL). The treatment with COMB­(II) and ESC significantly reduced QUIN levels in the hippocampus (21.30 ± 2.58 ng/mL; *p* < 0.01 and 23.60 ± 2.71; *p* < 0.05) compared to the Aβ_1–42_ + CMS group. However, the COMB­(I) and GAL exhibited nonsignificant changes in the QUIN levels (29.26 ± 2.41 ng/mL; *p* = 0.67 and 26.23 ± 1.65 ng/mL; *p* = 0.20), compared to the Aβ_1–42_ + CMS group (Figure 3C). One-way ANOVA for hippocampal QUIN levels revealed a large effect size (η^2^ = 0.46), indicating that treatment accounted for a substantial proportion of variance in neurotoxic metabolite accumulation. Thus, the therapy effectively reduced the concentrations of neurotoxic metabolites, 3-HK and QUIN, in the hippocampus of mice subjected to Aβ_1–42_ + CMS.

### 2.5 GAL and ESC Combination Reduced CORT Levels in the Alzheimer’s-Comorbid Depression Model

CORT is the glucocorticoid hormone in rodents, and it plays a crucial role in the stress response, energy metabolism, and modulation of brain function. The Aβ_1–42_ + CMS group showed a significant increase in the CORT levels in the plasma (73.15 ± 2.59 ng/mL; *p* < 0.01) compared to the sham group (51.90 ± 4.52 ng/mL). The treatment with COMB­(II) and ESC significantly lowered CORT levels in the plasma (57.62 ± 2.25 ng/mL, *p* < 0.05, and 57.43 ± 2.54 ng/mL, *p* < 0.05, respectively) compared to the Aβ_1–42_ + CMS group. In contrast, COMB­(I) and GAL exhibited nonsignificant changes in the CORT levels in the plasma (68.93 ± 2.89 ng/mL; *p* = 0.98 and 67.74 ± 2.46 ng/mL; *p* = 0.94), compared to the Aβ_1–42_ + CMS group (Figure 3D). One-way ANOVA for serum CORT levels revealed a large effect size (η^2^ = 0.49), indicating that treatment accounted for a substantial proportion of variance in stress hormone levels.

### 2.6 GAL and ESC Combination Alleviated AChE and BuChE Levels in the Alzheimer’s-Comorbid Depression Model

Aβ_1–42_ + CMS group significantly increased the AChE levels in the mouse hippocampus (0.27 ± 0.025 μmol/min/mg protein; *p* < 0.01) compared to the sham group (0.14 ± 0.020 μmol/min/mg protein). The treatment with COMB­(II) and GAL resulted in a decrease in the AChE levels (0.14 ± 0.007 μmol/min/mg protein; *p* < 0.01 and 0.17 ± 0.014 μmol/min/mg protein; *p* < 0.05), compared to the Aβ_1–42_ + CMS group. However, COMB­(I) and ESC exhibited nonsignificant changes in AChE levels (0.22 ± 0.03 μmol/min/mg protein; *p* = 0.68 and 0.19 ± 0.018 μmol/min/mg protein; *p* = 0.20), compared to the Aβ_1–42_ + CMS group (Figure 3E). One-way ANOVA for hippocampal AChE levels revealed a large effect size (η^2^ = 0.45), indicating that treatment accounted for a substantial proportion of variance in cholinergic activity.

The Aβ_1–42_ + CMS group significantly increased the butyrylcholinesterase enzyme (BuChE) levels in the mouse hippocampus (0.021 ± 0.002 μmol/min/mg protein; *p* < 0.01) compared to the sham group (0.011 ± 0.001 μmol/min/mg protein). The treatment with COMB­(II) and GAL resulted in a decrease in the BuChE levels (0.012 ± 0.0016 μmol/min/mg protein; *p* < 0.01 and 0.013 ± 0.0012 μmol/min/mg protein; *p* < 0.05), compared to the Aβ_1–42_ + CMS group. However, COMB­(I) and ESC exhibited nonsignificant changes in BuChE levels (0.017 ± 0.001 μmol/min/mg protein; *p* = 0.60 and 0.014 ± 0.0016 μmol/min/mg protein; *p* = 0.076), compared to the Aβ_1–42_ + CMS group (Figure 3F). One-way ANOVA for hippocampal BuChE levels revealed a large effect size (η^2^ = 0.48), indicating that treatment accounted for a substantial proportion of variance in cholinergic enzyme activity. The findings of the study highlighted the therapeutic relevance of GAL alone and combination therapy in modulating the concentration of AChE and BuChE for the management of AD-comorbid depression.

### 2.7 GAL and ESC Combination Ameliorated Proinflammatory Cytokines in the Alzheimer’s-Comorbid Depression Model

The Aβ_1–42_ + CMS group significantly increased the levels of IL-6 in the mouse hippocampus (46.53 ± 4.67 pg/mL; *p* < 0.05) compared to the sham group (26.39 ± 2.85 pg/mL). The treatment with COMB­(II) and ESC resulted in a decrease in IL-6 levels (28.17 ± 3.53; *p* < 0.05 and 28.14 ± 3.97; *p* < 0.05), respectively, compared to the Aβ_1–42_ + CMS group. However, COMB­(I) and GAL exhibited nonsignificant changes in IL-6 levels (39.06 ± 4.19; *p* = 0.89 and 35.34 ± 5.22; *p* = 0.52), compared to the Aβ_1–42_ + CMS group (Figure 4A). One-way ANOVA for hippocampal IL-6 levels revealed a large effect size (η^2^ = 0.45), indicating that treatment accounted for a substantial proportion of variance in the neuroinflammatory response.

**Figure 4:**
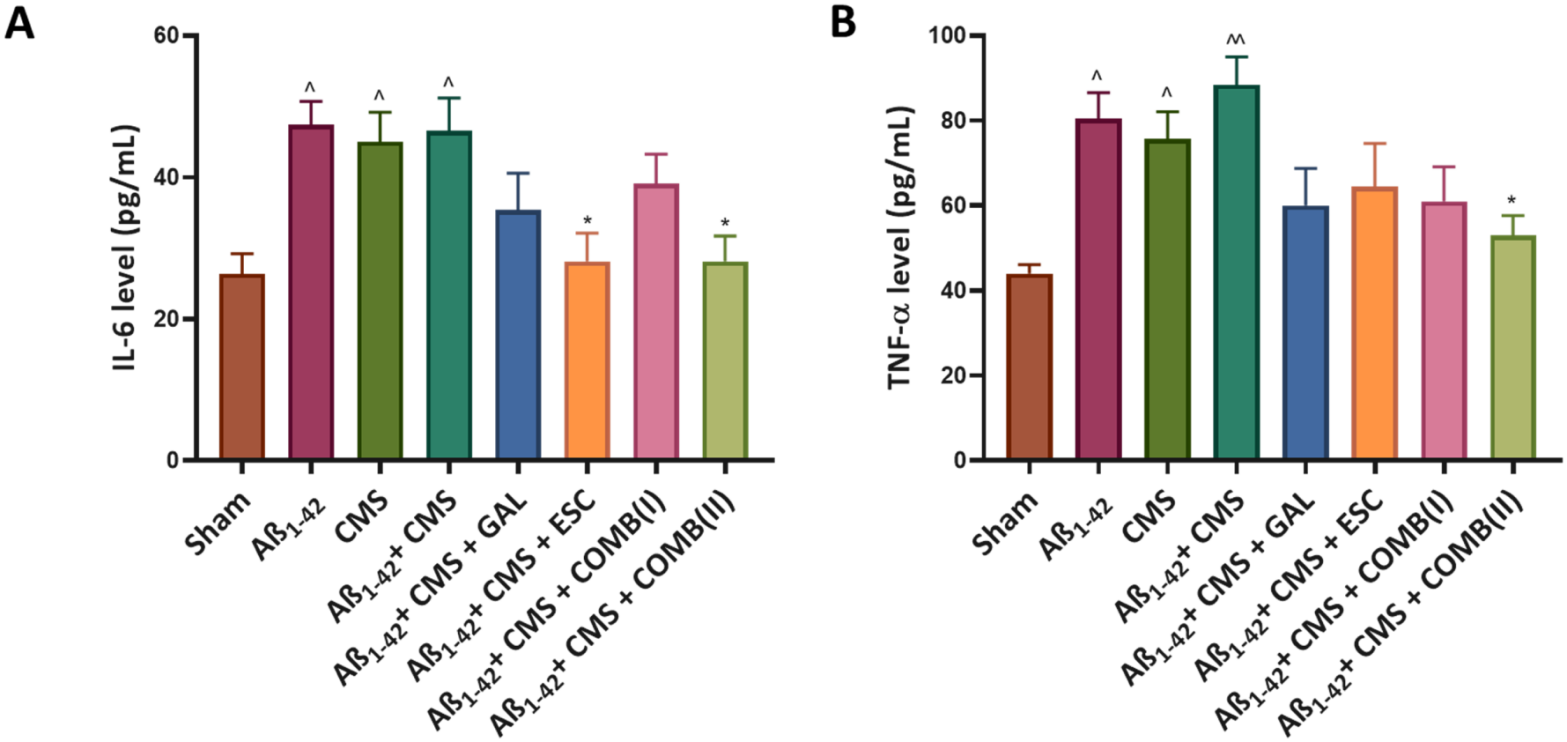
GAL, ESC, and combination normalize the levels of biochemical anomalies induced by the Aβ_1–42_ + CMS-induced model of AD-comorbid depression. A) IL-6 levels (pg/mL). B) TNF-α levels (pg/mL). The results were statistically analyzed by one-way ANOVA followed by the posthoc Tukey–Kramer multiple comparison test. The values were presented as mean ± SEM (*n* = 6). Statistical significance is marked as ^^*p* < 0.01, ^*p* < 0.05, compared to the sham group; **p < 0.01, and *p < 0.05, compared to the Aβ_1–42_ + CMS group. Doses: GAL (5 mg/kg), ESC (10 mg/kg), COMB­(I) (GAL; 3 mg/kg + ESC; 5 mg/kg), COMB­(II) (GAL; 5 mg/kg + ESC; 10 mg/kg).

The Aβ_1–42_ + CMS group significantly elevated TNF-α levels in the mouse hippocampus (88.42 ± 6.60 pg/mL; *p* < 0.01) compared to the sham group (44.08 ± 2.07 pg/mL). The treatment with COMB­(II) significantly reduced TNF-α levels in the mouse hippocampus (53.04 ± 4.55 pg/mL; *p* < 0.05) compared to the Aβ_1–42_ + CMS group. In contrast, COMB­(I), GAL, and ESC exhibited nonsignificant changes in the TNF-α levels (60.97 ± 8.13 pg/mL; *p* = 0.13, 60 ± 8.69 pg/mL; *p* = 0.10 and 64.38 ± 10.25 pg/mL; *p* = 0.25) , compared to the Aβ_1–42_ + CMS group (Figure 4B). One-way ANOVA for hippocampal TNF-α levels revealed a large effect size (η^2^ = 0.43), indicating that treatment accounted for a substantial proportion of variance in neuroinflammatory activity.

### 2.8 GAL and ESC Combination Alleviated Oxidative Stress in the Alzheimer’s-Comorbid Depression Model

The Aβ_1–42_ + CMS group exhibited increased oxidative stress, resulting in cellular damage to proteins, lipids, and DNA. In the current study, the Aβ_1–42_ + CMS group significantly elevated the levels of ROS, nitric oxide (NO), and malondialdehyde (MDA) in the hippocampus of mice compared to the sham group, as shown in Table 1. Monotherapy with ESC and GAL substantially normalizes the levels of oxidative stress markers. However, treatment with COMB­(II) exhibited a significant decrease in ROS, NO, and MDA levels compared to the Aβ_1–42_ + CMS group. In comparison, COMB­(I) exhibited nonsignificant changes in levels compared to the Aβ_1–42_ + CMS group. One-way ANOVA revealed large effect sizes for hippocampal ROS (η^2^ = 0.59), NO (η^2^ = 0.50), and MDA (η^2^ = 0.48) levels, indicating that treatment accounted for a substantial proportion of variance in oxidative stress markers.

**Table 1:** GAL, ESC, and Combination Reversed Oxidative Stress against Aβ_1–42_ + CMS-Induced Model of AD-Comorbid Depression^a^

S.No.	Groups	ROS level (AU)	NO (μM)	TBARS (*n* moles MDA/mg protein)	CAT (nmol/mg protein)	GSH (μg/mL)
1.	Sham	337360 ± 76494	34.67 ± 5.221	5.149 ± 0.7853	0.2033 ± 0.01308	3.329 ± 0.3219
2.	Aβ_1–42_	764625 ± 66465^^	74.02 ± 7.131^	12.94 ± 1.189^^	0.1433 ± 0.01229^	2.526 ± 0.2110
3.	CMS	856480 ± 31592^^^	79.93 ± 8.458^	11.31 ± 1.680^	0.1600 ± 0.01125	2.452 ± 0.1858
4.	Aβ_1–42_ + CMS	788295 ± 45147^^	94.14 ± 16.14^^^	13.28 ± 1.608^^	0.1483 ± 0.01249^	2.167 ± 0.2190^
5.	Aβ_1–42_ + CMS + GAL	449096 ± 76733*	51.50 ± 5.477*	7.249 ± 1.170*	0.1650 ± 0.01057	2.925 ± 0.08775
6.	Aβ_1–42_ + CMS + ESC	427090 ± 44755*	55.58 ± 4.044*	7.117 ± 1.389*	0.1783 ± 0.01249	2.890 ± 0.1598
7.	Aβ_1–42_ + CMS + COMB(I)	604689 ± 98320	78.49 ± 7.556	10.69 ± 0.9430	0.1633 ± 0.008819	2.616 ± 0.1995
8.	Aβ_1–42_ + CMS + COMB(II)	386882 ± 94914**	47.51 ± 7.125**	7.063 ± 1.509*	0.2000 ± 0.008563*	3.429 ± 0.3551**

a The results were statistically analyzed by one-way ANOVA and the posthoc Tukey–Kramer multiple comparison test; the values were presented as mean ± SEM (*n* = 6); statistical significance is marked as ^^^*p* < 0.001, ^^*p* < 0.01, ^*p*< 0.05, compared to the Sham group; **p < 0.01, and *p < 0.05, compared to the Aβ_1–42_ + CMS group; doses: GAL (5 mg/kg), ESC (10 mg/kg), COMB­(I) (GAL: 3 mg/kg + ESC: 5 mg/kg), and COMB­(II) (GAL: 5 mg/kg + ESC: 10 mg/kg).

In contrast, the Aβ_1–42_ + CMS group exhibited a decrease in the activity of antioxidant enzymes, including catalase (CAT) and glutathione (GSH), compared to the sham group (Table 1). The COMB­(II) treatment resulted in increased levels of CAT and GSH compared to the Aβ_1–42_ + CMS group. In comparison, COMB­(I), GAL, and ESC exhibited nonsignificant changes in the levels of antioxidant enzymes compared to the Aβ_1–42_ + CMS group. Overall, one-way ANOVA revealed large effect sizes for hippocampal CAT (η^2^ = 0.40) and GSH (η^2^ = 0.38) levels, indicating that treatment accounted for a substantial proportion of variance in antioxidant defenses.

### 2.9 GAL and ESC Combination Prevents Hippocampal Degeneration in Alzheimer’s- Comorbid Depression Model

Histopathological analysis of the hippocampus was performed using hematoxylin and eosin (H&E) staining to assess structural alterations in the CA1, CA3, and DG regions (Figure 5B). Mice exposed to Aβ_1–42_, CMS, or the combined Aβ_1–42_ + CMS insult exhibited pronounced structural degeneration in CA1, CA3, and DG compared to the sham group. In Figure 5B, black arrows denote degenerated neurons, whereas black dotted arrows indicate a loss of surrounding tissue. Treatment with GAL or ESC monotherapy attenuated hippocampal damage in CA1, CA3, and DG following Aβ_1–42_ + CMS exposure (Figure 5C–E). Notably, the combined therapy, particularly COMB­(II), produced a more robust neuroprotective effect, as evidenced by fewer degenerated neurons and preserved surrounding tissue in the CA1 and CA3 regions. Moreover, COMB­(II) preserved the structural integrity of the DG, maintaining the polymorphic layer and thereby safeguarding the hippocampal architecture and neuronal viability against neurodegenerative insults.

**Figure 5:**
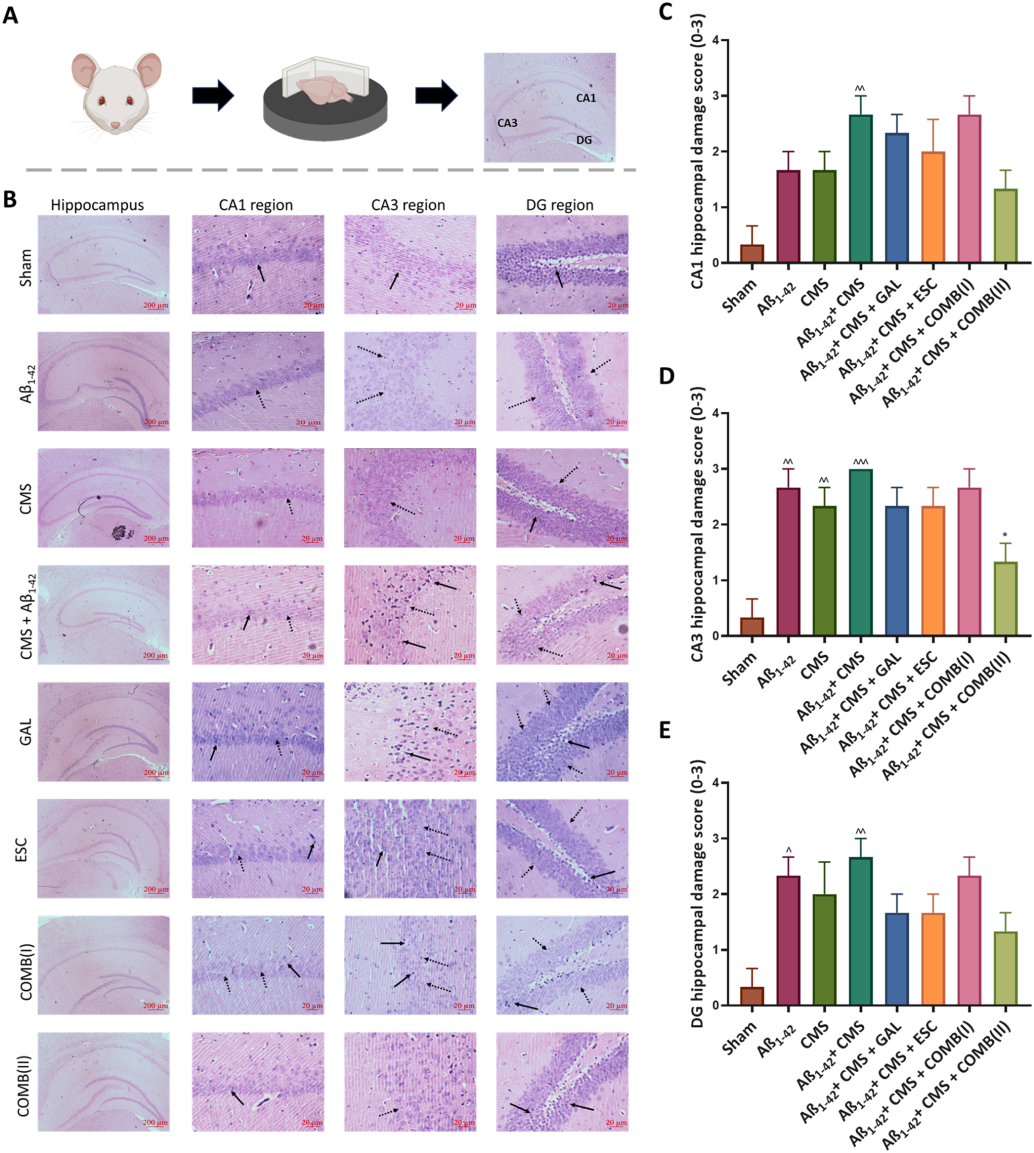
Histopathological analysis. A) Schematic diagram showing the histopathology study. B) H&E staining of the mice hippocampus. The black arrow indicates degenerated neurons, and the black dotted arrow shows the loss of the surrounding tissue (Scale bar: hippocampus; 200 μm and CA1, CA3, and DG region; 20 μm). Damage scores were assigned by a blinded observer across three hippocampal subfields: C) CA1 region, D) CA3 region, and E) DG region. A semiquantitative scoring system (0–3) was applied: 0 = intact hippocampal layers, 1 = mild disorganization, 2 = moderate layer disruption, 3 = severe layer loss. The results were statistically analyzed by one-way ANOVA followed by the posthoc Tukey–Kramer multiple comparison test. Statistical significance is marked as ^^^*p* < 0.001, ^^*p* < 0.01, and ^*p* < 0.05 compared to the sham group; *p < 0.05 compared to the Aβ_1–42_ + CMS group.

## 3 Discussion

Neurological comorbidity refers to the co-occurrence of a neuropsychiatric condition alongside a primary neurological disorder, potentially involving intricate relationships that complicate disease pathophysiology, management, or outcome. AD is commonly associated with various comorbidities and medical conditions, including depression, psychosis, sleep disturbances, epilepsy, hypertension, diabetes mellitus, atherosclerosis, and others. In particular, depression is the most commonly occurring psychiatric disorder observed in individuals with AD.^19^ Moreover, AD with comorbid depression represents a complex condition characterized by overlapping neurobiological abnormalities, including dysregulation of the neurotrophic system (BDNF), HPA axis (CORT), impaired kynurenine metabolism (elevated 3-HK and QUIN), altered inflammatory cytokine levels (TNF-α and IL-6), and disruptions in cholinergic neurotransmission (AChE and BuChE activity) and oxidative stress, all of which contribute to disease progression. Accordingly, the American Psychiatric Association recommends no pharmacotherapy for the treatment of AD-comorbid depression due to a lack of supportive data for drug-based effectiveness. The possible reason could be due to multiple pathways involved with AD and depression, including cellular machinery, protein accumulation, and neuronal loss.^3^ Given the lack of effectiveness, clinically, antidepressants belonging to the SSRIs class or AChE inhibitors are primarily prescribed as monotherapy. Due to the complexity of the comorbidity, monotherapy often fails to deliver remission in most clinical cases.^20^ To mitigate the shortcomings, combination therapy is frequently adopted to optimize pharmacological efficacy and minimize residual symptoms, ultimately improving the patient’s quality of life. Increased attention is being directed toward the combined use of SSRI agents and AChE inhibitors for managing AD with depression. This combined pharmacological approach is proposed to offer enhanced therapeutic benefits over standard monotherapy.

The tertiary alkaloid GAL, which is derived from *Galanthus nivalis*, is a competitive and reversible inhibitor of AChE and a positive modulator of α7 nAChR, and it is used to treat mild to moderate AD. GAL functions by increasing cholinergic neurotransmission, which is essential for maintaining cognition and preventing the breakdown of ACh in the synaptic cleft.^21^ ESC, the S (+) enantiomer of racemic citalopram, a highly potent SSRI that elevates serotonin levels at presynaptic neurons, is commonly used to treat depression in adults and adolescents. ESC alleviates neuroplasticity and hippocampal function, influences the HPA-axis, and normalizes neurotoxic entities associated with kynurenine metabolism, such as 3-HK and QUIN^22^ (Figure 6). This novel combination provides several advantages, including neuroprotection, multitargeted therapeutic action, enhanced cognitive function, and significant attenuation of inflammation and oxidative stress. Comprehensively, the study demonstrated the role of SSRIs (ESC) as a combination therapy with the AChE inhibitor (GAL) in a preclinical AD-comorbid depression model in mice, covering the neurobehavioral tests and histopathological and biochemical analyses involved in the disorder.

**Figure 6:**
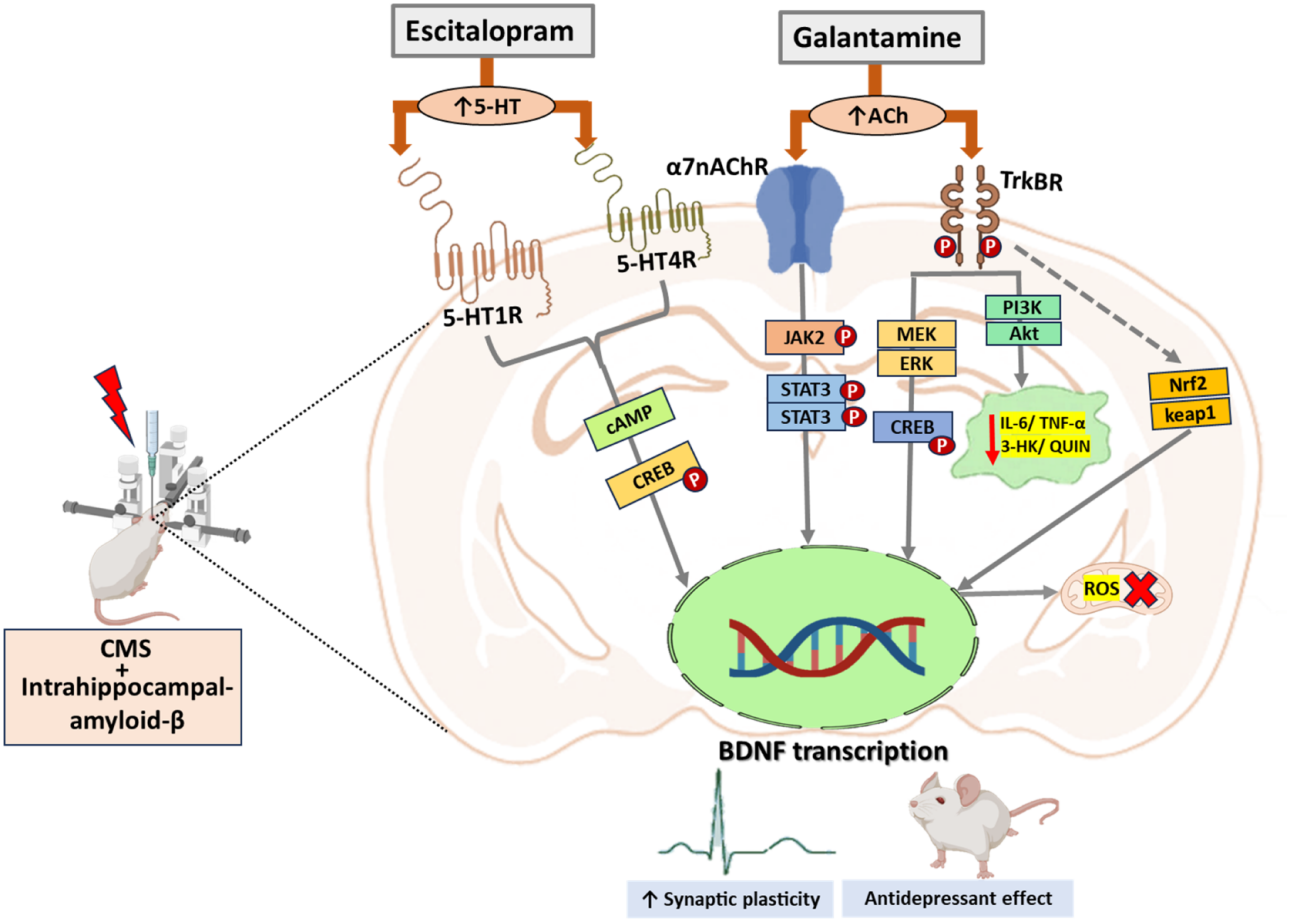
Probable mechanisms by which GAL and ESC exert neuroprotection against the Aβ_1–42_ + CMS-induced model of AD-comorbid depression. Abbreviations: 3-HK: 3-Hydroxykynurenine, 5-HT: Serotonin, 5-HT1R: Serotonin 1A receptor, 5-HT4R: 5-Hydroxytryptamine receptor 4, ACh: Acetylcholine, α7 nAChR: Alpha-7 nicotinic receptor, Akt: Protein kinase B, BDNF: Brain-derived neurotrophic factor, cAMP: Cyclic adenosine monophosphate, CREB: cAMP-response element binding protein, ERK: Extracellular-signal-regulated kinase, JAK2: Janus kinase 2, IL-6: Interleukin 6, IP3: Inositol trisphosphate, MEK: Mitogen-activated protein kinase, Nrf2: Nuclear factor erythroid 2-related factor 2, PI3K: Phosphatidylinositol 3-kinase, STAT3: Signal transducer and activator of transcription 3, TrkBR: Tyrosine receptor kinase B, TNF-α: Tumor necrosis factor-alpha, QUIN: Quinolinic acid.

The Aβ_1–42_ + CMS mouse model was employed to study AD-depression comorbidity. In the Aβ_1–42_ + CMS comorbid model, rodents were exposed to CMS for 3 weeks, followed by intrahippocampal injection of Aβ_1–42_ to mimic AD-like pathophysiology. The comorbid model exhibited physiological, neurobiological, and behavioral impairments that reflect overlapping pathological features characteristic of both AD and depression. The validity of the developed comorbid model was established by comparative behavioral and biochemical analyses against the Aβ_1–42_ and CMS single-exposure groups. The current study evaluated the combinational potential of GAL + ESC in the Aβ_1–42_ + CMS (AD and depression) comorbid mouse model. The outcome of combined therapy in alleviating the cognitive and depressive symptoms was assessed using different behavioral tests (including MWM, SAB, EPM, FST, and OFT), followed by histopathological and biochemical investigations. In this study, mice in the Aβ_1–42_ + CMS group exhibited pronounced depressive behavior and cognitive impairment compared to the sham group, as assessed through various neurobehavioral tests. Treatment with COMB­(II) significantly ameliorated depressive behavior relative to the Aβ_1–42_ + CMS group, whereas COMB­(I), GAL, and ESC monotherapies did not yield statistically significant effects. The improved outcome with combination therapy may be attributed to the additional benefits of GAL, which targets nAChRs in addition to the serotonergic modulation of ESC. Moreover, dual therapy enhances cholinergic neurotransmission, thereby indirectly modulating other neurotransmitter systems, including monoamines, glutamate, and GABA.^16,23^ Consistent with these findings, Zabot et al. reported that fluoxetine, fluoxetine + GAL, and fluoxetine + donepezil treatments effectively alleviated depressive-like behavior in a comorbid model, with the therapeutic effect being more pronounced in the combination therapy groups compared to the Aβ_1–42_ + CMS group.^17^

Moreover, cognitive impairment was observed in the Aβ_1–42_ + CMS comorbid model. Mice subjected to Aβ_1–42_ + CMS showed a significant decline in working and learning memory in SAB and MWM-based spatial tasks compared with the sham group. The investigation conducted by Srivareerat et al. aligns with our findings, reporting significant impairments in learning and memory following combined exposure to chronic stress and amyloid-β, as assessed using the radial arm water maze.^24^ Interestingly, treatment with COMB­(II), GAL, and ESC monotherapy resulted in a significant improvement in working and learning memory, as evidenced by increased % dwell time compared to the Aβ_1–42_ + CMS group, where dual therapy showed a stronger statistical association. The observed cognitive enhancement may be attributed to the complementary actions of the dual treatment; GAL restores cholinergic signaling and modulates α7 nAChR, while ESC enhances serotonergic tone and promotes neurogenesis. In addition, both agents modulate key molecular markers, including BDNF and synaptic proteins.^25–27^ The present study effectively investigated the role of cholinergic and serotonergic systems in mitigating memory impairment induced by dual insults of Aβ_1–42_ and CMS. Supporting the complementary nature of this therapeutic approach, Abe et al. evaluated the effects of RS-1259, a dual AChE and serotonin transporter inhibitor, in memory-impaired aged rats and demonstrated significant improvements in both working and learning memory.^28^ These findings strengthen our argument that simultaneous targeting of the serotonergic and cholinergic pathways offers therapeutic benefits, which can be clinically exploited to achieve greater therapeutic resilience.

Biochemical and histopathological parameters were investigated for GAL + ESC combination therapy in the Aβ_1–42_ + CMS comorbid model to understand the underlying mechanism of combined therapy in alleviating the disease pathophysiology. BDNF, a key neurotrophic factor, was quantified as it plays a critical role in regulating learning, memory, neuronal survival and differentiation, mood regulation, and the response to stress.^3^ Mice exposed to Aβ_1–42_ + CMS showed a decline in the levels of BDNF compared with the sham group, confirming the development of an AD comorbid depression model. These results align with the observations made by Rothman et al., who reported a decrease in BDNF levels in the triple-transgenic mouse model of AD (3xTg-AD mice) after exposure to chronic stress, which exacerbated impaired neurotrophic signaling.^29^ Interestingly, treatment with COMB­(II) as well as GAL and ESC monotherapies significantly increased BDNF levels compared to those of the Aβ_1–42_ + CMS group. Although monotherapies produced comparable improvements in BDNF, the complementary mechanisms and potential clinical relevance of the dual therapy underscore the importance of further investigating these two drugs in combination. Moreover, future studies should extend beyond BDNF to include other neurotrophic factors such as nerve growth factor (NGF) and glial cell line-derived neurotrophic factor (GDNF) to gain a more comprehensive understanding of their neuroprotective effects. The present findings are consistent with previous reports demonstrating BDNF elevation following both GAL and ESC treatment. For example, Ibrahim et al. investigated ESC in a D-galactose-injected ovariectomized rat model of AD and reported improved memory performance accompanied by enhanced hippocampal BDNF expression after 4 weeks of treatment.^30^ Similarly, Gil-Bea et al. examined GAL in a 192 IgG-saporin-induced model of cholinergic dysfunction, where the neurotoxin markedly reduced hippocampal BDNF expression. However, GAL treatment restored BDNF levels and ameliorated memory impairments in diseased rats.^31^

The kynurenine pathway is the other major contributing factor that plays a vital role in the implication and development of AD-depression comorbidity. Changes in metabolite levels can disrupt neurotransmitter signaling and the inflammatory profile, leading to neurotoxicity (Figure 6). In general, the kynurenine pathway maintains a balance between neuroprotective (kynurenine and kynurenic acid) and neurotoxic (3-HK and QUIN) metabolites, whose imbalance in levels can trigger disease progression.^32^ In the study, mice exposed to Aβ_1–42_ + CMS showed a significant increase in the levels of 3-HK and QUIN in the hippocampus compared with the sham group. The treatment with COMB­(II) resulted in a substantial decrease in the levels of 3-HK and QUIN compared to the Aβ_1–42_ + CMS group. These observations were supported by the findings of Fuertig et al., who reported that ESC monotherapy normalized the levels of 3-HK and kynurenic acid toward normal against the chronic social defeat-induced model of depression.^33^ In a similar context, Koola et al. demonstrated that administration of a GAL-memantine combination significantly decreased 3-HK levels in individuals with schizophrenia, indicating that AChE inhibitors may modulate the kynurenine pathway and attenuate neurotoxicity.^34^ The likely mechanism underlying modulation of the kynurenine pathway by ESC + GAL therapy is twofold: ESC indirectly influences indoleamine 2,3-dioxygenase (IDO), reducing its activity and thereby shifting tryptophan metabolism away from neurotoxic metabolites, whereas GAL directly facilitates α7 nAChR activity to regulate the kynurenine pathway. The resultant normalization of kynurenine signaling contributes to neuroprotective effects.^35,36^

Furthermore, the HPA-axis function is a neurological pathway highly affected by AD and depression. Chronic stress overactivates the HPA-axis, causing an elevation in CORT levels. High levels of CORT are associated with increased levels of amyloid precursor protein processing and amyloid-β production. Abnormal CORT levels are related to synaptic dysfunction, dendritic atrophy, oxidative stress, and inflammation.^37^ In the study, mice exposed to Aβ_1–42_ + CMS exhibited a significant increase in the levels of CORT in plasma compared with the sham group. The treatment with COMB­(II) and ESC monotherapy significantly lowered CORT levels compared to the Aβ_1–42_ + CMS group. In the AD-comorbid depression model, ESC and GAL modulate CORT levels through complementary mechanisms. ESC normalizes HPA-axis activity via serotonergic regulation of corticotropin-releasing hormone (CRH) and glucocorticoid receptor (GR)-mediated negative feedback, whereas GAL indirectly influences HPA-axis function by attenuating inflammation. Together, their combination synergistically restores HPA homeostasis and confers neuroprotective effects.^12,38^ Previous reports support these findings. Doron et al. demonstrated that CORT levels were elevated in an unpredictable CMS-induced model of depression, and treatment with ESC effectively normalized circulating CORT.^39^ Similarly, GAL reduced CORT levels in a lipopolysaccharide-induced model of neuroinflammation, highlighting the potential of AChE inhibitors in modulating HPA-axis activity.^40^

AD and depression are known to disrupt cholinergic neurotransmission. A significant loss of cholinergic neurons in the basal nucleus is thought to be positively associated with both conditions.^3^ AChE and BuChE are the key enzymes responsible for the hydrolysis of ACh in the synaptic cleft, thereby regulating cholinergic neurotransmission. In AD, depression, and other mood disorders, the activity of AChE and BuChE is often dysregulated, leading to excessive breakdown of ACh and exacerbation of cholinergic deficits. Cholinesterase inhibitors, including dual AChE and BuChE inhibitors, enhance synaptic ACh availability, improving cognitive function and behavioral outcomes. Mice exposed to Aβ_1–42_ + CMS exhibited a significant increase in hippocampal AChE and BuChE levels compared to the sham group, wherein the treatment with COMB­(II) and GAL alone showed a significant decrease in AChE and BuChE compared to the Aβ_1–42_ + CMS group. Interestingly, ESC monotherapy was found to reduce AChE and BuChE levels, although these effects did not reach statistical significance. The reduction in cholinesterase activity observed with ESC suggests a possible interplay between depressive pathology and cholinergic dysfunction in the comorbid condition. This observation highlights the need for further studies to delineate the mechanistic link between depressive behavior and cholinergic signaling, as such interactions may contribute to the worsening of cognitive deficits in AD with comorbid depression. Moreover, the findings align with those of Walsh et al., who examined the combined effect of GAL and citalopram on the kinetic behavior of BuChE inhibition. The combined treatment produced a significant inhibition of BuChE, which was synergistic and facilitated cholinergic neurotransmission.^9^ In a comparable study, Abe et al. investigated the effects of RS-1259, a dual AChE and serotonin transporter inhibitor, in an aged rat model of AD. The treatment significantly elevated hippocampal ACh levels, and the dual inhibition of AChE and the serotonin transporter markedly improved memory performance in aged rats.^28^

In neurological disorders, inflammation is a hallmark contributing to disease progression. However, whether inflammation is a cause or a consequence of neurobiological alterations remains unclear. Inflammation is a common pathological feature shared by both AD and depression, playing a significant role in cognitive decline, hippocampal degeneration, neurotransmitter imbalance, impaired neuroplasticity, blood–brain barrier dysfunction, and hyperactivation of the immune response.^41^ The mice exposed to Aβ_1–42_ + CMS exhibited a significant increase in IL-6 and TNF-α levels in the hippocampus compared with the sham group. The findings were in accordance with those of Zabot et al., wherein proinflammatory cytokines, IL-6 and TNF-α, were elevated postexposure to Aβ_1–42_ + CMS. However, treatment with fluoxetine + donepezil and fluoxetine + GAL significantly reversed the levels of these inflammatory markers in the hippocampus of comorbid rats.^17^ Treatment with COMB­(II) produced a significant reduction in IL-6 and TNF-α levels compared with the Aβ_1–42_ + CMS group. Notably, ESC monotherapy also attenuated IL-6 levels, underscoring its potential in counteracting neuroinflammation. The marked decline in proinflammatory cytokines with COMB­(II) may be attributed to its complementary mechanisms of action, wherein the dual therapy simultaneously engages multiple pathways, including neurotrophic signaling, HPA-axis regulation, neurotransmitter modulation, normalization of kynurenine metabolism, and mitigation of oxidative stress. Collectively, these converging effects contribute to a more robust anti-inflammatory response, thereby supporting the therapeutic advantage of combinatorial treatment in AD with comorbid depression. In agreement, Benatti et al. demonstrated the antineuroinflammatory effects of ESC in a chronic escape deficit model of depression, where ESC treatment significantly reduced hypothalamic proinflammatory cytokines, including IL-6 and IL-1β, highlighting its neuroprotective potential.^42^ Similarly, Liu et al. reported the anti-inflammatory efficacy of GAL in a lipopolysaccharide-induced model of neuroinflammation. Mice treated with GAL for 14 days exhibited marked reductions in hippocampal levels of IL-1β, IL-6, and TNF-α, suggesting its potential as a therapeutic agent for attenuating neuroinflammation. These findings reinforce the notion that ESC and GAL act on complementary yet overlapping inflammatory pathways, providing a mechanistic rationale for the enhanced efficacy of their combined use.^27^

Furthermore, sustained inflammation contributes directly to mitochondrial dysfunction and the overproduction of free radicals in both AD and depression. As a result, oxidative stress represents a prominent pathological mechanism under these conditions. An imbalance between the generation of ROS and the antioxidant defense system leads to cellular damage. This damage is particularly exacerbated in cases of comorbidity, promoting a self-perpetuating cycle of neurodegeneration.^19^ In a study, mice exposed to Aβ_1–42_ + CMS insults exhibited reduced levels of CAT and GSH (antioxidant enzymes) and increased levels of ROS, NO, and MDA (oxidative species) compared to the sham group. The oxidative stress observed in the dual-insult model may be attributed to impaired Nrf2-mediated antioxidant defense, NF-κB-driven inflammatory signaling, mitochondrial ROS overload, and toxicity arising from kynurenine pathway dysregulation.^43,44^ The COMB­(II) treatment significantly alleviated oxidative stress compared to Aβ_1–42_ + CMS in the hippocampus (ROS, 2.03-fold; NO, 1.98-fold; MDA, 1.88-fold), indicating its antioxidant potential. Interestingly, the monotherapies ESC and GAL were able to modulate oxidative markers such as ROS, NO, and MDA; however, no statistically significant changes were observed in the levels of CAT and GSH. These findings suggest that dual therapy may exert complementary effects in counteracting oxidative imbalance under comorbid conditions. Previously, the findings of Matchkov et al. support these observations, demonstrating that ESC treatment significantly attenuated oxidative stress in a CMS-induced depression model. The therapy effectively restored GSH and MDA levels in the rat brain, underscoring the potential of SSRI-based interventions in mitigating oxidative damage.^45^ Kadian et al. consistently investigated the effect of GAL in reversing oxidative stress markers against the intracerebroventricular streptozotocin-induced model of AD. The treatment significantly reversed alterations in ROS, SOD, GSH, CAT, MDA, and NO levels in the rat’s hippocampus, underscoring its antioxidant potential.^46^ Although GAL and ESC primarily act as neurotransmitter modulators, their receptor-mediated signaling cascades can activate intrinsic antioxidant defenses through the Nrf2, BDNF, and PI3K/Akt pathways, while also modulating inflammation, metabolically active tissues, and kynurenine metabolites, ultimately contributing to the reduction of oxidative stress in the brain.^47^

In AD and depression, the hippocampus is the central region impacted along with disease progression and marked explicitly with neuronal loss, impaired memory, and reduced hippocampal volume. A histopathological study was carried out to analyze the structural changes in the hippocampus. Mice exposed to Aβ_1–42_, CMS, and Aβ_1–42_ + CMS showed degenerated neurons, loss of surrounding tissue at CA1 and CA3 regions, and disruption of the polymorphic layer at the DG region compared to the sham group. However, hippocampal damage was significantly more prominent in the Aβ_1–42_ + CMS group. GAL or ESC monotherapy treatment prevented morphological damage induced by Aβ_1–42_ + CMS insults; the protective effects were less pronounced than those observed with the combined therapy. Furthermore, the combined therapy, especially COMB­(II), demonstrated a marked improvement in hippocampal structure with fewer degenerated neurons and intact surrounding tissue in the CA1, CA3, and DG regions compared to the Aβ_1–42_ + CMS group. Overall, the combined treatment maintained the hippocampal structure, indicating a pronounced neuroprotective effect. These histological findings align with the behavioral and biochemical outcomes, wherein COMB­(II) not only preserved hippocampal structural integrity but also enhanced BDNF expression, reduced neuroinflammation and oxidative stress, and restored HPA-axis balance. Together, these converging effects highlight the capacity of combined ESC and GAL therapy to provide comprehensive neuroprotection against dual insults of Aβ_1–42_ and chronic stress, thereby improving both functional and structural outcomes in AD with comorbid depression. Supporting these findings, Ibrahim et al. reported pyknosis and necrosis of pyramidal neurons in the hippocampus of ovariectomized rats, a widely used model of depression. Treatment with ESC, 17-β estradiol, and their combination notably preserved the hippocampal architecture more effectively and reduced the number of necrotic neurons, thereby offering protection against pathological insults.^48^ Similarly, Ganainy et al. investigated the effects of GAL, oxytocin, and their combination on hippocampal structure in an aluminum chloride-induced model of AD. Rats exposed to aluminum chloride exhibited marked hippocampal damage, which was ameliorated following treatment with GAL, oxytocin, or their combination.^49^ Notably, the combined therapy significantly reversed structural damage in the hippocampus, with pyramidal cells appearing to be well-preserved and orderly arranged.

In conclusion, AD is a complex neurological disorder associated with cognitive dysfunction and related affective symptoms, including depression, anxiety, sleep disturbances, and psychosocial disabilities. To date, there is no approved therapy for managing AD with comorbid depression, and monotherapy with either AChE inhibitors or SSRIs has demonstrated limited efficacy in addressing overlapping symptoms. The combined therapeutic approach offers a promising intervention to treat AD with comorbid depression and to overcome monotherapy-related limitations, such as a poor therapeutic response, increased risk of adverse effects, and drug tolerance. Among combination strategies, the concurrent use of SSRI agents and AChE inhibitors has shown potential. Therefore, the present study investigated the combined use of GAL and ESC in a CMS- and Aβ_1–42_-induced mouse model of AD comorbid depression. The combined treatment with GAL and ESC resulted in a significant improvement in depressive symptoms and cognitive dysfunction, exceeding the effects of monotherapy. Furthermore, the combination therapy had a positive influence on neurotrophic factors (BDNF), kynurenine pathway metabolites (3-HK/QUIN), neuroinflammation (TNF-α and IL-6), and oxidative stress markers in the hippocampus of mice with comorbid diseases. Importantly, the combined treatment reduced neuronal degeneration and preserved the structural integrity of the hippocampus against CMS + Aβ_1–42_ insult. These findings highlight the clinical translational potential of ESC and GAL combination therapy in managing AD with comorbid depression.

The present study acknowledges certain limitations. Stereotaxic injection of Aβ_1–42_ reproduces key features of Aβ-induced synaptic dysfunction and cognitive impairment. When combined with CMS, it highlights plausible mechanisms such as HPA axis activation, BDNF depletion, and neuroinflammation through which depression may accelerate AD-like pathology. The comorbid model employed in this study was adapted from previously published work and provides valuable insights into shared neurobiological mechanisms.^17,24,29,50,51^ Nevertheless, because this approach represents an acute, nonprogressive insult and does not account for aging or other common comorbidities, the findings should be viewed as mechanistic and hypothesis-generating rather than as direct surrogates of the slowly progressive nature of human AD. Another limitation of the present study is that, although animals of both sexes were randomly distributed across groups with balanced representation to minimize sex-related confounding, sex-specific differences were not systematically analyzed. Considering the well-documented influence of sex on stress reactivity, neuroinflammation, and antidepressant response, future studies should explicitly address sex-dependent variations to provide a more comprehensive understanding of treatment efficacy in AD comorbid with depression.

Although the combination therapy (ESC + GAL) did not demonstrate statistically significant superiority over monotherapy in direct group-to-group comparisons, it produced numerically greater effects, relative to disease controls. Considering ESC’s role in promoting serotonergic synaptic plasticity and GAL’s cholinergic enhancement, the two agents are likely to act in a complementary manner. The absence of statistical superiority may be attributed to potential ceiling effects or the limited treatment duration required to elicit additional benefits. Nevertheless, the complementary mechanisms of ESC and GAL highlight their possible clinical relevance, warranting further investigation in extended preclinical and clinical settings. A deeper molecular understanding of the comorbid pathophysiology is essential to elucidate the underlying mechanisms and bridge the gap between preclinical findings and clinical manifestations of the disease.

## 4 Materials and Methods

### 4.1 Drugs and Chemicals

Escitalopram oxalate (purity >98%) and galantamine hydrobromide (purity >98%) were procured from Tokyo Chemical Industry (TCI), India Ltd. Aβ_1–42_ was obtained from Abcam Ltd., UK. Mouse Enzyme-Linked Immunosorbent Assay (ELISA) kits to estimate BDNF, CORT, QUIN, 3-HK, and cytokines were procured from Elabscience, USA, or Krishgen, India. The remaining reagents and chemicals used were of analytical grade and were procured from local vendors.

### 4.2 *In Vivo* Experimental Procedure

The study used Swiss albino mice of either sex, weighing between 25 and 30 g and aged 10–12 weeks. The mice were obtained from the central animal facility (BITS Pilani, Pilani campus, Rajasthan) after approval from the Institutional Animal Ethics Committee (IAEC) with protocol number IAEC/RES/34/13/REV-01/36/20. Animals in groups of four were housed in polypropylene cages at a temperature of 25 ± 1 °C, a relative humidity of 50–70%, and a controlled 12 h light–dark cycle, with an environment that provided free access to food and water *ad libitum,* as per the standard protocol. All experiments complied with the Committee for the Control and Supervision of Experiments on Animals (CCSEA), India, and Animal Research: Reporting of *In* *Vivo* Experiments (ARRIVE) guidelines.

#### 4.2.1 Chronic Mild Stress-Induced Depression-Like Pathology in Mice

CMS is a well-established animal model of depression that involves the application of a series of mild, unpredictable, noninvasive stressors over a sustained period to induce depression-like symptoms in rodents. The behavioral abnormalities observed following CMS include anhedonia, learned helplessness, and reduced locomotor activity. The CMS protocol was based on previous methodology with slight modifications.^52^ The sham group was not exposed to any CMS-based manipulation throughout the 21-day procedure and was allowed access to food and water *ad libitum*. The CMS group was exposed to unpredictable stressors according to the schedule mentioned for 21 days, as shown in Table 2. The development of depressive symptoms was confirmed randomly across cages using OFT and EPM paradigms, as per the established laboratory guidelines. After the confirmation of depressive symptoms, from day 22 onward, animals were injected with Aβ_1–42_ (amyloid-β; neurotoxic peptide) using stereotactic surgery (detailed protocol mentioned in section 4.2.2). After recovery from the surgery, animals were continued with the CMS regime simultaneously with the treatment for 20 days to determine the effectiveness of the therapy.

**Table 2:** Schedule for the CMS Procedure

S No.	Stressors	Duration
Day 1	Food/water deprivation	12 h
Day 2	Empty bottles	1 h
	Foreign object	12 h
Day 3	Forced swimming	4 min/animal
	Overnight illumination	10 h
Day 4	Restraint (80 cm^3^ breathable, transparent, plastic enclosing)	1 h
	Cage tilt	5 h
Day 5	Food deprivation	12 h
	Wet sawdust	12 h
Day 6	Water deprivation	12 h
	Overnight illumination	12 h
Day 7	Flashing light (stroboscopic light, 50 flashes/min)	3 h
	Cage tilt (250^°^)	5 h

#### 4.2.2 Amyloid-β_1–42_-Induced Alzheimer’s Model Using Stereotactic Surgery in Mice

Swiss albino mice were anesthetized intraperitoneally with ketamine (80 mg/kg) and xylazine (10 mg/kg) and placed in the stereotaxic apparatus. Then, eye ointment was applied to prevent corneal drying. The skulls of the mice were shaved, cleaned, and incised to locate the intrahippocampal coordinates: −1.7 mm, ±1.8 mm, and −2.0 mm, respectively, at anteroposterior (AP), medial-lateral (ML), and dorsoventral (DV) directions relative to the bregma and ventral direction from the dura, with the tooth bar set at 0 mm. Bilateral holes were carefully drilled using a microdriller to expose the brain. To confirm the accuracy of the stereotaxic coordinates, trypan blue dye was injected in a pilot animal at the target site and verified postmortem. To induce the AD pathology in the animals, the oligomeric form of Aβ_1–42_ was administered as per previously reported literature.^53–55^ Each animal was infused with a total of 400 pmol of Aβ_1–42_ bilaterally on the dorsal hippocampus at a flow rate of 0.5 μL/min using a 28-gauge Hamilton microsyringe with a volume of 1.5 μL/site. Similarly, the sham group underwent the same procedure with an infusion of phosphate-buffered saline (PBS) without Aβ_1–42_. To prevent backflow after injection, the syringe was kept undisturbed at the site for 5 min. A drilled hole in the skull was filled using bone wax, and the incised region was sutured and kept for observation. During surgery, mice were placed on a heating pad to prevent hypothermia. Postsurgery, a single analgesic dose was administered subcutaneously, and mice were placed in a heated recovery chamber and were continuously monitored until they regained consciousness. Daily monitoring was performed to assess for signs of infection or pain in the animals until the wound had completely healed.

#### 4.2.3 *In Vivo* Treatment

An AD comorbid depression model was developed in mice, wherein the animals were randomly divided into eight groups of 12 mice per group. The sample size was determined to be 12 animals per treatment group using G*Power software (version 3.1.9.7), based on the following parameters: type I error (α) = 0.05, power (1−β) = 0.95, effect size (*d*) = 1.06, and an allocation ratio (N_2_/N_1_) = 1. The power analysis was conducted using a *t* test for comparison of means between the two groups. The effective doses for GAL, ESC, combination COMB­(I), and COMB­(II) were selected based on previous studies.^42,56–59^ The treatment groups include Sham, Aβ_1–42_ (AD control), CMS (depression control), Aβ_1–42_ + CMS (diseased; comorbid control), GAL (5 mg/kg), ESC (10 mg/kg), COMB­(I) (GAL; 3 mg/kg + ESC; 5 mg/kg), and COMB­(II) (GAL; 5 mg/kg + ESC; 10 mg/kg), respectively. The drug treatment was continued for 20 days after recovery from stereotactic surgery. All drug solutions were freshly prepared and administered orally, as both ESC and GAL exhibit good absorption and bioavailability via this route. Moreover, since both agents are clinically prescribed in humans through oral administration, the use of the same route in this study enhances the translational relevance of the findings. The study initially included a predetermined number of animals in each group; however, mortality was observed across diseased groups. Mortality was evident in the Aβ_1–42_ group with 16% mortality, the CMS group with 8% mortality, with the highest rate occurring in the Aβ_1–42_ + CMS with 25% mortality. The Sham group did not exhibit any mortality during the study period. Additionally, the Aβ_1–42_ + CMS group treated with GAL (16% mortality) and COMB­(I) (16% mortality) exhibited some degree of mortality. Surprisingly, ESC and COMB­(II) showed 8% mortality during the study period. To account for this attrition and ensure reliable outcomes, sample sizes were matched across groups. The final sample sizes were adjusted as follows: behavioral assessments were included with 8 animals per group, biochemical analyses with 6 animals per group, and histopathological evaluations with 3 animals per group. The exclusion of animals was carried out in accordance with established laboratory protocols, primarily based on predefined criteria such as morbid or unhealthy animals that could not be ethically or reliably continued in the study. These steps were taken to maintain consistency and scientific validity across all experimental groups. All procedures were performed in accordance with established laboratory protocols to ensure consistency and adherence to ethical standards. After completion of the study, mice were humanely euthanized, and brain tissue and plasma were collected. Brain tissue from all groups was snap-frozen and stored at −80 °C for biochemical evaluation according to the manufacturer’s protocol. Remaining brain samples from each group were stored in 4% formalin for histopathological analysis.

#### 4.2.4 Behavioral Analysis

##### 4.2.4.1 Depression-Specific Behavioral Analysis (FST, OFT, and EPM)

EPM comprises two open and two closed arms, a standard test for anxiety-like behavior. Based on arm preference, mice were allowed to explore the maze freely, and thereafter, the depressed state of the animals was calculated.^60^ Subsequently, OFT was used to evaluate the exploratory activity in rodents, in which the behavioral state of the mice was measured based on the time spent at the center or corners of the arena.^61^ FST was conducted using the procedure described by Porsolt, in which mice were placed into a glass cylinder filled with water, and the duration of immobility was measured.^62^ All behavioral assessments were conducted by an observer blinded to the treatment groups to ensure unbiased evaluation. The behavioral tests were performed and recorded in accordance with established protocols and analyzed using Stoelting Any-maze software (version 7.44, USA). Detailed experimental procedures are provided in Supplementary File S1.

##### 4.2.4.2 Cognition-Specific Behavioral Analysis (SAB and MWM)

SAB^63^ and MWM tests^64^ were used to evaluate rodent cognitive function and working memory. In the SAB, mice were placed on the X-maze’s central platform and allowed to explore freely. The alternation of each animal was calculated and noted. A % decrease in the alternation was described as cognitive impairment. Furthermore, habituation, acquisition, and probe trials were conducted in a circular stainless-steel tank with controlled lights for the MWM test. The time required by the mouse to locate the hidden platform was measured as the escape latency, followed by the calculation of the path length and average speed. On the last day of the test, a probe trial was performed to assess reference memory and learning. Recorded movements were analyzed using Stoelting-AnyMaze software (version 7.44, USA). All behavioral assessments were conducted by an observer blinded to the treatment groups to ensure unbiased evaluation. The behavioral tests were performed and recorded in accordance with established protocols and analyzed using Stoelting-AnyMaze software (version 7.44, USA). Detailed experimental procedures are provided in Supplementary File S1.

#### 4.2.5 Biochemical Parameters

##### 4.2.5.1 Brain Tissue Homogenate Preparation

The hippocampal tissue from the whole brain of mice was isolated and rinsed with ice-cold saline and then minced and homogenized in PBS (pH 7.4) at a 1:10 (w/v) ratio using a glass homogenizer under ice-cold conditions. The homogenate was centrifuged at 10,000 rpm at 4 °C for 10 min, and the supernatant was collected. The samples were stored at −80 °C until further use.

##### 4.2.5.2 Measurement of BDNF, 3-HK, and QUIN in the Mouse Hippocampus

The quantification of BDNF in the mouse hippocampus was estimated using a mouse-specific ELISA kit (Elabscience, USA) according to the manufacturer’s protocol. Briefly, 100 μL/well of standard and test samples were added to the plate and incubated for 1.5 h at 37 °C. The plate was aspirated, and 100 μL of biotinylated antibody solution was added to each well and incubated for 1 h at 37 °C. Then, the solution was decanted from each well and washed four times with wash buffer. Thereafter, 100 μL of streptavidin-HRP solution was added to each well and incubated for another 30 min at 37 °C. Again, the solution was decanted from each well and washed with wash buffer. Lastly, 90 μL of TMB substrate solution was added, and the mixture was incubated for 15 min under dark conditions, followed by the addition of 50 μL of stop solution to each well. The absorbance readings were acquired at 450 nm in a Biotek plate reader, and the data were plotted. Similarly, levels of 3-HK and QUIN were estimated according to the manufacturer’s protocol (detailed protocols are provided in Supporting Information File S1).

##### 4.2.5.3 Estimation of CORT in the Plasma

Determination of CORT levels in mouse plasma was estimated using a mouse-specific ELISA kit (Elabscience, USA) according to the manufacturer’s protocol. Briefly, 50 μL of standard and test samples were added to the plate, followed by the addition of 50 μL of biotinylated antibody solution to each well, and the plate was then incubated for 45 min at 37 °C. Then, the solution from each well was decanted and washed four times with wash buffer. Thereafter, 100 μL of streptavidin-HRP solution was added to each well, and the mixture was incubated for another 30 min at 37 °C. The solution from each well was decanted and washed with the wash buffer. Lastly, 90 μL of TMB substrate solution was added and incubated for 15 min under dark conditions, followed by the addition of 50 μL of stop solution to each well. The absorbance readings were acquired at 450 nm by using a Biotek plate reader, and the data were plotted.

##### 4.2.5.4 Measurement of Cholinesterase Activity (AChE and BuChE) in Mice Hippocampus

The increase in cholinesterase enzyme activity is associated with reduced ACh levels due to hydrolysis. Enzyme activity was measured using Ellman’s assay. The principle of this assay is based on the formation of thiocholine from acetylcholine iodide and butyrylthiocholine iodide by AChE and BuChE, respectively. Thiocholine reacts with Ellman’s reagent (5,5′-dithiobis­(2-nitrobenzoic acid) or DTNB) to produce a yellow-colored product, which can be measured spectrophotometrically at 412 nm. For AChE estimation, the reaction mixture comprised 50 μL of acetylcholine iodide (10 mM), 50 μL of buffered Ellman’s reagent DTNB (10 mM in 15 mM NaHCO_3_), and 140 μL of PBS (0.1 M, pH 7.4). Similarly, for BuChE estimation, the reaction mixture comprised 50 μL of butyrylthiocholine iodide (10 mM), 50 μL of buffered Ellman’s reagent DTNB (10 mM in 15 mM NaHCO_3_), and 140 μL of PBS (0.1 M, pH 7.4). The mixture was incubated at 37 °C for 10 min, after which 10 μL of hippocampal homogenate was added. The optical density was measured at 412 nm within 5 min using a BioTek spectrophotometer. AChE and BuChE activities were expressed as μmol of substrate hydrolyzed per minute per milligram of protein (μmol/min/mg protein).^65^

##### 4.2.5.5 Analysis of Pro-Inflammatory Cytokine Expression (IL-6 and TNF-α) in Mice Hippocampus

IL-6 and TNF-α levels were estimated using a mouse-specific ELISA kit according to the manufacturer’s protocol (detailed protocols are provided in Supporting Information File S1).

##### 4.2.5.6 Oxidative Stress Markers (CAT, NO, ROS, MDA, GSH)

Enzymatic assays of thiobarbituric acid-reactive substances (TBARS)/MDA,^66^ ROS,^67^ NO,^68^ GSH,^69^ and CAT^70^ were measured spectrophotometrically in a Biotek plate reader according to the procedure described previously (detailed protocols are mentioned in Supplementary File S1).

##### 4.2.5.7 Protein Estimation

The total protein concentration in the hippocampus was estimated using the Lowry et al. method, with bovine serum albumin (BSA) as a standard^71^ (detailed protocols are provided in Supplementary File S1).

#### 4.2.6 Histopathology Study

Animals were sacrificed after behavioral studies, and the brains were excised for histopathological examination. Structural features of the hippocampal regions, including CA1, CA3, and DG, were analyzed using H&E staining. The stained sections were evaluated for the integrity of surrounding tissue and pyramidal cells, signs of neuronal degeneration, and the condition of the polymorphic layer in the DG region. A blinded observer performed the histopathological scoring of different hippocampal regions. Microscopic images were captured using an inverted microscope, the Axio Vert. A1 FL-LED (ZEISS), and data were analyzed using the Zen Blue software (version 3.4).

## 5 Statistical Analysis

Data are presented as mean ± SEM. Statistical analyses were performed using GraphPad Prism version 8.0.2 (GraphPad Inc., CA, USA). Repeated-measures ANOVA was used for MWM parameters (escape latency, path length, and swimming speed), while one-way ANOVA followed by Tukey’s posthoc test was applied to all other measures. Mean differences were reported with 95% confidence intervals. Effect sizes (η^2^ for one-way ANOVA and partial η^2^ for two-way ANOVA) were calculated from ANOVA outputs, where η^2^ = SSeffect/SStotal and ηp^2^ = SSeffect/(SSeffect + SSerror).

## Supplementary Material



## Notes

Experimental procedures for behavioral and biochemical assays (PDF)

## Abbreviations

AD: Alzheimer’s disease
AChE: acetylcholinesterase enzyme
ACh: acetylcholine
α7 nAChRs: α7 nicotinic acetylcholine receptors
AP: anteroposterior
Aβ_1–42_: amyloid beta
BDNF: brain-derived neurotrophic factor
BSA: bovine serum albumin
CAT: catalase
CMS: chronic mild stress
CORT: Corticosterone
DV: dorsoventral
ESC: escitalopram
EPM: elevated plus maze
FST: forced swim test
GAL: galantamine
GABA: gamma-aminobutyric acid
GSH: glutathione
H&E: hematoxylin and eosin
HPA: hypothalamic–pituitary–adrenal
IL-6: interleukin-6
MDA: malondialdehyde
MWM: Morris water maze
ML: medial-lateral
NMDA: *N*-methyl-d-aspartate
NO: nitric oxide
OFT: open field test
PBS: phosphate buffer saline
QUIN: quinolinic acid
ROS: reactive oxygen species
SSRIs: selective serotonin reuptake inhibitors
SNRIs: serotonin-norepinephrine reuptake inhibitors
SAB: spontaneous alternation behavior
TNF-α: tumor necrosis factor alpha
3-HK: 3-hydroxykynurenine
DTNB: 5,5′-dithiobis­(2-nitrobenzoic acid)

## References

[1] World Health Organization. Dementia. https://www.who.int/news-room/fact-sheets/detail/dementia (accessed 29/09).

[2] Zhao Q.-F., Tan L., Wang H.-F., Jiang T., Tan M.-S., Tan L., Xu W., Li J.-Q., Wang J., Lai T.-J. (2016). The
prevalence of neuropsychiatric symptoms in Alzheimer’s disease:
systematic review and meta-analysis. J. Affective
Disord.

[3] Bajaj S., Mahesh R. (2024). Converged avenues:
depression and Alzheimer’s
disease–shared pathophysiology and novel therapeutics. Mol. Biol. Rep..

[4] Spina S., Joie R. L., Petersen C., Nolan A. L., Cuevas D., Cosme C., Hepker M., Hwang J.-H., Miller Z. A., Huang E. J. (2021). Comorbid
neuropathological diagnoses in early
versus late-onset Alzheimer’s disease. Brain.

[5] Kaur D., Sharma V., Deshmukh R. (2019). Activation of microglia and astrocytes:
a roadway to neuroinflammation and Alzheimer’s disease. Inflammopharmacology.

[6] Vyas S., Rodrigues A. J., Silva J. M., Tronche F., Almeida O. F. X., Sousa N., Sotiropoulos I. (2016). Chronic stress
and glucocorticoids:
from neuronal plasticity to neurodegeneration. Neural Plast..

[7] Liang Y., Xie S., He Y., Xu M., Qiao X., Zhu Y., Wu W. (2022). Kynurenine pathway
metabolites as biomarkers in Alzheimer’s
disease. Dis. Markers.

[8] Burke A. D., Goldfarb D., Bollam P., Khokher S. (2019). Diagnosing and treating
depression in patients with Alzheimer’s disease. Neurol. Ther..

[9] Walsh R., Rockwood K., Martin E., Darvesh S. (2011). Synergistic
inhibition
of butyrylcholinesterase by galantamine and citalopram. Biochim. Biophys. Acta.

[10] Toda N., Tago K., Marumoto S., Takami K., Ori M., Yamada N., Koyama K., Naruto S., Abe K., Yamazaki R. (2003). A conformational
restriction approach to the
development of dual inhibitors of acetylcholinesterase and serotonin
transporter as potential agents for Alzheimer’s disease. Bioorganic Med. Chem..

[11] Zarra J., Schmidt L. (2010). Mild cognitive disorder and depression:
treatment with
combination of galantamine and escitalopram. Annals Gen. Psychiatry.

[12] Ago Y., Koda K., Takuma K., Matsuda T. (2011). Pharmacological aspects
of the acetylcholinesterase inhibitor galantamine. J. Pharmacol. Sci..

[13] Millan M. J., Agid Y., Brüne M., Bullmore E. T., Carter C. S., Clayton N. S., Connor R., Davis S., Deakin B., DeRubeis R. J. (2012). Cognitive
dysfunction in psychiatric disorders:
characteristics, causes and the quest for improved therapy. Nat. Rev. Drug Discovery.

[14] Koola M. M. (2018). Attenuated
mismatch negativity in attenuated psychosis syndrome predicts psychosis:
can galantamine-memantine combination prevent psychosis?. Complex Psychiatry.

[15] Yin J., Song X., Wang C., Lin X., Miao M. (2023). Escitalopram
versus other antidepressive agents for major depressive disorder:
a systematic review and meta-analysis. BMC Psychiatry.

[16] Philip N. S., Carpenter L. L., Tyrka A. R., Price L. H. (2010). Nicotinic acetylcholine
receptors and depression: a review of the preclinical and clinical
literature. Psychopharmacology.

[17] Zabot G. C., Medeiros E. B., Macarini B. M. N., Peruchi B. B., Keller G. S., Lídio A. V., Boaventura A., de Jesus L. C., de Bem
Silveira G., Silveira P. C. L., Chede B. C., Réus G. Z., Budni J. (2024). The involvement of neuroinflammation in an animal model of dementia
and depression. Prog. Neuro-Psychopharmacol.
Biol. Psychiatry.

[18] Koola M. M., Nikiforuk A., Pillai A., Parsaik A. K. (2018). Galantamine-memantine
combination superior to donepezil-memantine combination in Alzheimer’s
disease: critical dissection with an emphasis on kynurenic acid and
mismatch negativity. J. Geriatr. Care Res..

[19] Maciejewska K., Czarnecka K., Szymański P. (2021). A review of the mechanisms underlying
selected comorbidities in Alzheimer’s disease. Pharmacol. Rep..

[20] Baruch N., Burgess J., Pillai M., Allan C. L. (2019). Treatment for depression
comorbid with dementia. BMJ. Ment Health.

[21] Kita Y., Ago Y., Higashino K., Asada K., Takano E., Takuma K., Matsuda T. (2014). Galantamine promotes adult hippocampal neurogenesis
via M1 muscarinic and α 7 nicotinic receptors in mice. Int. J. Neuropsychopharmacol..

[22] Malki K., Lourdusamy A., Binder E., Payá-Cano J., Sluyter F., Craig I., Keers R., McGuffin P., Uher R., Schalkwyk L. C. (2012). Antidepressant-dependent
mRNA changes
in mouse associated with hippocampal neurogenesis in a mouse model
of depression. Pharmacogenetics Genomics.

[23] Dulawa S. C., Janowsky D. S. (2019). Cholinergic regulation of mood: from basic and clinical
studies to emerging therapeutics. Mol. Psychiatry.

[24] Srivareerat M., Tran T. T., Alzoubi K. H., Alkadhi K. A. (2009). Chronic psychosocial
stress exacerbates impairment of cognition and long-term potentiation
in β-amyloid rat model of Alzheimer’s disease. Biol. Psychiatry.

[25] Johansen A., Armand S., Plavén-Sigray P., Nasser A., Ozenne B., Petersen I. N., Keller S. H., Madsen J., Beliveau V., Møller K. (2023). Effects of
escitalopram on synaptic
density in the healthy human brain: A randomized controlled trial. Mol. Psychiatry.

[26] Jorge R. E., Acion L., Moser D., Adams H. P., Robinson R. G. (2010). Escitalopram
and enhancement of cognitive recovery following stroke. Arch. Gen. Psychiatry.

[27] Liu Y., Zhang Y., Zheng X., Fang T., Yang X., Luo X., Guo A., Newell K. A., Huang X. -F., Yu Y. (2018). Galantamine
improves cognition, hippocampal inflammation, and synaptic plasticity
impairments induced by lipopolysaccharide in mice. J. Neuroinflammation.

[28] Abe Y., Aoyagi A., Hara T., Abe K., Yamazaki R., Kumagae Y., Naruto S., Koyama K., Marumoto S., Tago K. (2003). Pharmacological characterization
of RS-1259, an orally
active dual inhibitor of acetylcholinesterase and serotonin transporter,
in rodents: possible treatment of Alzheimer’s disease. J. Pharmacol. Sci..

[29] Rothman S. M., Herdener N., Camandola S., Texel S. J., Mughal M. R., Cong W.-N., Martin B., Mattson M. P. (2012). 3xTgAD mice exhibit
altered behavior and elevated Aβ after chronic mild social stress. Neurobiol. Aging.

[30] Ibrahim W. W., Abdelkader N. F., Ismail H. M., Khattab M. M. (2019). Escitalopram Ameliorates
Cognitive Impairment in D-Galactose-Injected Ovariectomized Rats:
Modulation of JNK, GSK-3β, and ERK Signalling Pathways. Sci. Rep..

[31] Gil-Bea F. J., Solas M., Mateos L., Winblad B., Ramírez M. J., Cedazo-Mínguez A. (2011). Cholinergic
hypofunction impairs
memory acquisition possibly through hippocampal Arc and BDNF downregulation. Hippocampus.

[32] Jacobs K. R., Lim C. K., Blennow K., Zetterberg H., Chatterjee P., Martins R. N., Brew B. J., Guillemin G. J., Lovejoy D. B. (2019). Correlation between plasma and CSF
concentrations of
kynurenine pathway metabolites in Alzheimer’s disease and relationship
to amyloid-β and tau. Neurobiol. Aging.

[33] Fuertig R., Azzinnari D., Bergamini G., Cathomas F., Sigrist H., Seifritz E., Vavassori S., Luippold A., Hengerer B., Ceci A., Pryce C. R. (2016). Mouse chronic social stress increases
blood and brain kynurenine pathway activity and fear behaviour: Both
effects are reversed by inhibition of indoleamine 2,3-dioxygenase. Brain, Behav., Immun..

[34] Koola M. M., Sklar J., Davis W., Nikiforuk A., Meissen J. K., Sawant-Basak A., Aaronson S. T., Kozak R. (2018). Kynurenine
pathway in schizophrenia: Galantamine-memantine combination for cognitive
impairments. Schizophr Res..

[35] Abd
El-Fattah E. E. (2022). IDO/kynurenine pathway in cancer: possible therapeutic
approaches. J. Transl. Med..

[36] Frost E. D., Shi S. X., Byroju V. V., Rissardo J. P., Donlon J., Vigilante N., Murray B. P., Walker I. M., McGarry A., Ferraro T. N. (2024). Galantamine-Memantine
combination in the treatment
of parkinson’s disease dementia. Brain
Sci..

[37] Dong H., Yuede C. M., Yoo H. S., Martin M. V., Deal C., Mace A. G., Csernansky J. G. (2008). Corticosterone
and related receptor
expression are associated with increased beta-amyloid plaques in isolated
Tg2576 mice. Neuroscience.

[38] Bhatt S., Devadoss T., Jha N. K., Baidya M., Gupta G., Chellappan D. K., Singh S. K., Dua K. (2023). Targeting inflammation:
a potential approach for the treatment of depression. Metab. Brain Dis..

[39] Doron R., Lotan D., Versano Z., Benatav L., Franko M., Armoza S., Kately N., Rehavi M. (2014). Escitalopram or novel
herbal mixture treatments during or following exposure to stress reduce
anxiety-like behavior through corticosterone and BDNF modifications. PLoS One.

[40] Pavlov V. A., Parrish W. R., Rosas-Ballina M., Ochani M., Puerta M., Ochani K., Chavan S., Al-Abed Y., Tracey K. J. (2009). Brain acetylcholinesterase
activity controls systemic cytokine levels through the cholinergic
anti-inflammatory pathway. Brain, Behav., Immun..

[41] Ly M., Yu G. Z., Mian A., Cramer A., Meysami S., Merrill D. A., Samara A., Eisenstein S. A., Hershey T., Babulal G. M., Lenze E. J., Morris J. C., Benzinger T. L. S., Raji C. A. (2023). Neuroinflammation: A Modifiable Pathway
Linking Obesity, Alzheimer’s disease, and Depression. Am. J. Geriatric Psychiatry.

[42] Benatti C., Alboni S., Blom J. M. C., Mendlewicz J., Tascedda F., Brunello N. (2018). Molecular changes associated
with
escitalopram response in a stress-based model of depression. Psychoneuroendocrinology.

[43] Gella A., Durany N. (2009). Oxidative stress in
Alzheimer disease. Cell Adhesion Migration.

[44] Bhatt S., Nagappa A. N., Patil C. R. (2020). Role of oxidative stress in depression. Drug Discovery Today.

[45] Matchkov V. V., Kravtsova V. V., Wiborg O., Aalkjaer C., Bouzinova E. V. (2015). Chronic
selective serotonin reuptake inhibition modulates endothelial dysfunction
and oxidative state in rat chronic mild stress model of depression. Am. J. Physiol..

[46] Kadian M., Saini N., Khera A., Kumar A. (2024). Neuroprotective mechanism
of trans,trans-Farnesol in an ICV-STZ-induced rat model of Alzheimer’s
pathology. Inflammopharmacology.

[47] Koola M. M., Praharaj S. K., Pillai A. (2019). Galantamine-memantine combination
as an antioxidant treatment for schizophrenia. Curr. Behav. Neurosci. Rep..

[48] Ibrahim W. W., Safar M. M., Khattab M. M., Agha A. M. (2016). 17β-Estradiol
augments antidepressant efficacy of escitalopram in ovariectomized
rats: Neuroprotective and serotonin reuptake transporter modulatory
effects. Psychoneuroendocrinology.

[49] El-Ganainy S. O., Soliman O. A., Ghazy A. A., Allam M., Elbahnasi A. I., Mansour A. M., Gowayed M. A. (2022). Intranasal
Oxytocin Attenuates Cognitive
Impairment, β-Amyloid Burden and Tau Deposition in Female Rats
with Alzheimer’s Disease: Interplay of ERK1/2/GSK3β/Caspase-3. Neurochemi. Res..

[50] Shlomi-Loubaton S., Nitzan K., Rivkin-Natan M., Sabbah S., Toledano R., Franko M., Bentulila Z., David D., Frenkel D., Doron R. (2025). Chronic stress leads
to earlier cognitive decline in an Alzheimer’s
mouse model: The role of neuroinflammation and TrkB. Brain, Behav., Immun..

[51] Lin L.-Y., Zhang J., Dai X.-M., Xiao N.-A., Wu X.-L., Wei Z., Fang W.-T., Zhu Y.-G., Chen X.-C. (2016). Early-life stress
leads to impaired spatial learning and memory in middle-aged ApoE4-TR
mice. Mol. Neurodegener..

[52] Kurhe Y., Mahesh R. (2016). Pioglitazone, a PPARγ agonist
rescues depression
associated with obesity using chronic unpredictable mild stress model
in experimental mice. Neurobiol. Stress.

[53] Arriagada J., Merceron D., Ardiles A., Muñoz P., Paula-Lima A. (2025). Excitatory-inhibitory synaptic imbalance
induced by
acute intra-hippocampus injections of amyloid-β oligomers. Biochem. Biophys. Res. Commun..

[54] Fa M., Orozco I. J., Francis Y. I., Saeed F., Gong Y., Arancio O. (2010). Preparation of oligomeric β-amyloid1–42
and induction of synaptic plasticity impairment on hippocampal slices. J. Visualized Exp..

[55] Chen S., Guo D., Zhu Y., Xiao S., Xie J., Zhang Z., Hu Y., Huang J., Ma X., Ning Z. (2024). Amyloid
β oligomer induces cerebral vasculopathy via pericyte-mediated
endothelial dysfunction. Alzheimer’s
Res. Ther..

[56] Bajaj S., Mahesh R. (2025). Galantamine as add-on
therapy to escitalopram: enhancing
antidepressant therapeutic potential via. Targeting α7nAChR/BDNF/KYN
signalling. Psychopharmacology.

[57] Bhagya V., Srikumar B., Raju T., Shankaranarayana
Rao B. (2011). Chronic escitalopram
treatment restores spatial learning, monoamine levels, and hippocampal
long-term potentiation in an animal model of depression. Psychopharmacology.

[58] Saito T., Hisahara S., Iwahara N., Emoto M. C., Yokokawa K., Suzuki H., Manabe T., Matsumura A., Suzuki S., Matsushita T. (2019). Early administration
of galantamine from preplaque phase suppresses oxidative stress and
improves cognitive behavior in APPswe/PS1dE9 mouse model of Alzheimer’s
disease. Free Radical Biol. Med..

[59] Nozaki S., Hijioka M., Wen X., Iwashita N., Namba J., Nomura Y., Nakanishi A., Kitazawa S., Honda R., Kamatari Y. O. (2024). Galantamine suppresses
α-synuclein aggregation
by inducing autophagy via the activation of α7 nicotinic acetylcholine
receptors. J. Pharmacol. Sci..

[60] Walf A. A., Frye C. A. (2007). The use of the elevated
plus maze as an assay of anxiety-related
behavior in rodents. Nat. Protoc..

[61] Wang S., Huang G., Yan J., Li C., Feng J., Chen Q., Zheng X., Li H., Li J., Wang L. (2021). Influence of aging on chronic unpredictable
mild stress-induced
depression-like behavior in male C57BL/6J mice. Behav. Brain Res..

[62] Porsolt R. D., Le Pichon M., Jalfre M. (1977). Depression: a new animal model sensitive
to antidepressant treatments. Nature.

[63] Vohora D., Pal S. N., Pillai K. (2005). Modulation
of spontaneous alternation
performance of mice treated with thioperamide and tacrine in a cross
maze task. Fundamental Clin. Pharmacol..

[64] Vorhees C. V., Williams M. T. (2006). Morris water maze: procedures for assessing spatial
and related forms of learning and memory. Nat.
Protoc..

[65] Ellman G. L., Courtney K. D., Andres V., Featherstone R. M. (1961). A new and
rapid colorimetric determination of acetylcholinesterase activity. Biochemi. Pharmacol..

[66] Ohkawa H., Ohishi N., Yagi K. (1979). Assay for
lipid peroxides in animal
tissues by thiobarbituric acid reaction. Anal.
Biochem..

[67] Niknahad H., Heidari R., Mohammadzadeh R., Ommati M. M., Khodaei F., Azarpira N., Abdoli N., Zarei M., Asadi B., Rasti M. (2017). Sulfasalazine induces
mitochondrial dysfunction and renal injury. Ren. Fail..

[68] Wu D., Yotnda P. (2011). Production and detection
of reactive oxygen species
(ROS) in cancers. J. Visualized Exp..

[69] Hissin P. J., Hilf R. (1976). A fluorometric method for determination of oxidized and reduced glutathione
in tissues. Anal. Biochem..

[70] Sinha A. K. (1972). Colorimetric
assay of catalase. Anal. Biochem..

[71] Lowry O. H., Rosebrough N. J., Farr A. L., Randall R. J. (1951). Protein measurement
with the Folin phenol reagent. J. Biol. Chem..

