# Supplementary material for: Galantamine–Escitalopram Combination Therapy in Alzheimer’s Comorbid Depression Model in Mice: Role of BDNF/KYN Pathways, Neuroinflammation, and Oxidative Stress
Source: ACS Omega. 2026 Jan 28;11(5):7396–414. doi: 10.1021/acsomega.5c08276 (PMC12902959; doi:10.1021/acsomega.5c08276)
Supplement: Supplementary file 1 [file ao5c08276_si_001.pdf]

Supplementary Information

Galantamine-Escitalopram Combination Therapy in Alzheimer's Comorbid Depression Model in Mice: Role of BDNF/KYN Pathways, Neuroinflammation, and Oxidative Stress

Shivanshu Bajaj [a], Radhakrishnan Mahesh [a]*

[a] Department of Pharmacy, Birla Institute of Technology & Science (BITS), Pilani Campus, Pilani-333031, Rajasthan, India.

*Email: rmahesh@pilani.bits-pilani.ac.in

Behavioural Assays

Elevated plus-maze (EPM)

EPM test was used to measure the anxiety-like behaviours in mice, as reported previously. The dimension of the apparatus consists of two open arms and two closed arms; each having dimensions of 36 cm × 6 cm × 15 cm (length × width × height) and raised 60 cm from the floor. During the test, mice were placed individually in the centre of the apparatus facing towards the open arm and were allowed to freely explore the maze for 5 min. After each session, the maze was cleaned with a 70% alcohol solution. Time spent (seconds; s) in the open arm and closed arm was measured for 5 min with the Stoelting-Any-maze software (version 7.44, IL, USA).

Open field test (OFT)

The OFT test was used to measure the exploratory activities in rodents. The mice were kept individually in the centre of the arena of dimensions (70 × 70 × 42 cm; l × b × h), and movement was tracked for 10 min. After each session, the apparatus was cleaned with a 70% alcohol solution to eliminate any olfactory interference. Recorded movements were analysed using the Stoelting-Any-maze software (version 7.44, IL, USA).

Forced swim test (FST)

FST was conducted as described by Porsolt et al. with some modifications. Mice were individually placed into the glass cylinder with a height and diameter of 35 and 15 cm, respectively, filled with water, and the temperature was maintained at 25 ± 1 °C. The test was carried out for 6 min, for which the total duration of immobility was determined after a 2-minute pre-test period. Therefore, immobility was measured for 4 minutes. The animals were said to be immobile once they were floating passively.

Spontaneous alternation behaviour (SAB)

SAB behaviour was calculated using the previously reported protocol with slight modifications. SAB was calculated in a cross maze of dimension 23.5 × 8 × 10 cm (l × b × h) and raised 50 cm above the floor to prevent the animal from escaping. During the experiment, mice were gently placed individually on the central platform and were allowed to freely explore the maze. The test was conducted for 6 min/animal. After each session, the apparatus was cleaned with a 70% alcohol solution to eliminate any olfactory interference. Throughout the experiment, the number of sequences of each arm was video recorded. Alternation was defined as entry into four different arms on overlapping quintuple sets. The percentage of alternation was calculated by the following formula.

$$\%\, alternation = \frac{actual\ alternation}{possible\ alternation} \times 100$$

Actual alternation represented the total number of quintuple sets, which comprised a set of five consecutive arm choices within the total set of arm choices, while possible alternation was equal to the total number of arm entries minus 4.

Morris Water Maze (MWM) test

MWM was comprised of a large circular stainless-steel tank filled with water, with dimensions 120 cm in diameter and 50 cm in height and had a non-reflecting surface. A circular plexiglass platform/island (12 cm in diameter) was submerged at a level 0.5-1 cm below the surface of a water-filled tank. The platform was fixed and made completely invisible as per the background. Water temperature was maintained at 25 ± 2 °C to avoid hypothermia in animals, and other conditions, such as light and sound, were controlled to prevent errors. To establish spatial learning and memory, surrounding cues of different shapes were mounted in each quadrant on the tank wall for mice to follow to reach the platform.

For spatial acquisition, the tank was virtually divided into four equal quadrants using two principal axes named east, west, north and south. The escape platform was located in one quadrant (North-West) and remained fixed throughout the experiment. In the task, every mouse underwent four session trials with an inter-trial interval of 30 s continuously for 5 days. Every mouse was positioned opposite the pool wall and allowed to freely navigate the spatial environment to find the hidden platform within a ceiling time of 60 s. During each trial, mice were allowed to sit on the platform for 30 s to get familiar with the surrounding and cues. In cases where the mouse failed to escape to the platform within the stipulated time of 60 s, it was guided gently to the hidden platform and allowed to remain there for the trial duration. In each trial, escape latency, which was the time the mouse took to allocate to the hidden platform, and path length, which was the distance travelled by mouse to reach the hidden platform, were noted for 5 consecutive days.

On the 6th day, a probe trial was performed to assess reference memory. For this, the hidden platform was removed from the MWM tank, and the mouse was placed into the water maze in the novel quadrant (opposite to the initial platform location). Mice were allowed to swim freely for 60 s. The dwell time, which was the time spent by the mouse in the target quadrant (where the platform was positioned earlier), was recorded, and it represents the consolidated memory followed by learning.

Estimation of 3-HK

The quantification of 3-HK in mice hippocampus was estimated using a mouse-specific ELISA kit (Krishgen Biosystems, India) per the manufacturer's protocol. Briefly, 40 µL/well of standard and test samples were added to the plate and immediately added with 10 µL biotinylated antibody solution and 50 µL of streptavidin-HRP solution. Mix well. Cover the plate and incubate for 1 h at 37 °C. The plate was aspirated and washed four times with wash buffer. Lastly, 100 µL of TMB (3,3',5,5'-Tetramethylbenzidine) substrate solution was added and incubated for 10 min under dark conditions, followed by 100 µL of stop solution in each well. The absorbance readings were acquired

at 450 nm, and the data were plotted.

Estimation of QUIN

The quantification of QUIN in mice hippocampus was estimated using a mouse-specific ELISA kit (Krishgen Biosystems, India) per the manufacturer's protocol. Briefly, 50 µL/well of standard and test samples were added to the plate, immediately added with 50 µL biotinylated antibody solution, and incubated for 1 h at 37 °C. Then, decant the solution from each well and wash it four times with a wash buffer. Thereafter, 100 µL of streptavidin-HRP solution was added to each well and incubated for another 1 h at 37 °C. Again, decant the solution from each well and wash it with a wash buffer. Lastly, 100 µL of TMB substrate solution was added and incubated for 10 min under dark conditions, followed by 100 µL of stop solution in each well. The absorbance readings were acquired at 450 nm, and the data were plotted.

Estimation of IL-6

The quantification of IL-6 was estimated using a mouse-specific ELISA kit (Krishgen Biosystems, India) per the manufacturer's protocol. Briefly, 100 µL/well of standard and test samples were added to the plate and incubated for 2 h at room temperature. The plate was aspirated and washed four times with wash buffer. Then 100 µL of biotinylated antibody solution was added to each well and incubated for 1 h at room temperature. Again, decant the solution from each well and wash it with a wash buffer. Thereafter, 100 µL of streptavidin-HRP solution was added to each well and incubated for another 1 h at 37 °C. Again, decant the solution from each well and wash it with a wash buffer. Lastly, 100 µL of TMB substrate solution was added and incubated for 30 min under dark conditions, followed by 100 µL of stop solution in each well. The absorbance readings were acquired at 450 nm, and the data were plotted.

Estimation of TNF-α

The quantification of TNF-α was estimated using a mouse-specific ELISA kit (Krishgen Biosystems, India) per the manufacturer's protocol. Briefly, 100 µL/well of standard and test samples were added to the plate and incubated for 2 h at 37 °C. The plate was aspirated and washed four times with wash buffer, and 100 µL of biotinylated antibody solution was added to each well and incubated for 1 h at 37 °C. Then, decant the solution from each well and wash it four times with a wash buffer. Thereafter, 100 µL of streptavidin-HRP solution was added to each well and incubated for another 30 min at 37 °C. Again, decant the solution from each well and wash it with a wash buffer. Lastly, 100 µL of TMB substrate solution was added and incubated for 30 min under dark conditions, followed by 100 µL of stop solution in each well. The absorbance readings were acquired at 450 nm, and the data were plotted.

Oxidative Stress Markers:

Catalase (CAT) enzyme assay:

CAT concentrations were calculated as nmol/mg of protein. Briefly, the enzymatic activity of CAT was calculated based on the principle that in the presence of H_2O_2, dichromate in acetic acid is reduced to chromic acetate, which was measured calorimetrically. In this method, 150 µL of reaction mixture was prepared, which consists of 10 µL test sample, 100 µL of 0.01M phosphate buffer, and 40 µL of 2M H_2O_2. Later, adding 200 µL of dichromacetic acid reagent (prepared by adding 5% w/v potassium dichromate and glacial acetic acid in a 1:3 ratio) stopped the reaction. Absorbance was recorded at 530 nm, and data were plotted.

Measurement of nitric oxide (NO) levels:

The NO level in tissue samples was measured per the protocol. Briefly, 50 µL of sample was added with 50 µL of 1% w/v Sulphanilamide solution and incubated for 10 min at room temperature in the dark. Then 50 µL of 0.1% w/v N-1-napthylethylenediamine dihydrochloride solution was added and mixed well. Incubate the mixture for 10 min under dark conditions, and the absorbance was immediately measured with a wavelength between 520 nm and 550 nm. NO concentrations were calculated as µM.

Reactive oxygen species (ROS) assay:

ROS concentration was quantified as per the protocol. In brief, 100 µL of tissue homogenate was incubated with 1 mL of 2’,7’-dichlorofluorescein diacetate (10 µM) for 40 min at 37 °C. Tissue autofluorescence was measured in control, 100 µL of tissue homogenate incubated with 1 mL of Tris-HCl buffer under similar conditions. The fluorescence intensity of the samples was assessed using a BioTek plate reader with excitation and emission wavelengths of 485 and 525 nm.

Thiobarbituric acid-reactive substances (TBARS):

Initially, 200 µL of tissue homogenate was mixed with 200 µL of sodium dodecyl sulphate (SDS) solution (8.1% w/v). After that, 1.5 mL of 20% acetic acid solution (pH 3.5) and 1.5 mL of 0.8% thiobarbituric acid solution were added. Subsequently, distilled water was added to the mixture to bring its volume up to 4 mL, and it was heated for 1 h at 95 °C. After cooling, the tubes were vigorously shaken before adding 1 mL of distilled water and 5 mL of n-butanol and pyridine mixture (15:1, v/v). Centrifugation was performed for 10 min at 4000 rpm to separate the organic layer. At 532 nm, the organic layer's absorbance was measured compared to the blank sample. MDA concentrations were calculated as nmoles of MDA/mg of protein.

Reduced Glutathione (GSH) assay:

GSH is in a reduced state and is one of the most prevalent antioxidant defence components in tissue. In summary, mix 100 µL of tissue homogenate with 1.8 mL of phosphate-EDTA buffer, pH 8. Then add 100 µL of o-phthalaldehyde solution, mix it thoroughly, and incubate at room temperature for 15 min. Using a BioTek fluorescent plate reader with excitation and emission wavelengths of 355

and 420 nm, the fluorescent signal generated by the specific reaction of o-phthalaldehyde with GSH at pH 8 was measured. GSH concentrations were calculated as μg/mL.

Protein estimation:

The total protein concentration in the hippocampus was determined using Lowry et al.'s method, with bovine serum albumin serving as the standard. In brief, 50 µl of hippocampal homogenate or standard was mixed with 225 µL of Reagent I (2% Na_2CO_3 in 0.1N NaOH, 1% sodium potassium tartrate, and 0.5% $CuSO_4$) and incubated for 10 min in the dark. Subsequently, 25 µL of Reagent II (1:1 mixture of Folin-Phenol reagent and water) was added, and the mixture was incubated for 30 min in the dark. The absorbance was then measured at 660 nm using a BioTek spectrophotometer, and the data were plotted.
